# Synthesis of Poly(butylene adipate-*co*-terephthalate) with Branched Monomer for Biodegradable Copolyesters with Enhanced Processability and Rheological Properties

**DOI:** 10.1021/acsomega.5c00277

**Published:** 2025-04-04

**Authors:** Xinpeng Zhang, Hongli Bian, Xiangze Meng, Jing Yuan, Jianping Ding, Wanli Li, Jun Xu, Baohua Guo

**Affiliations:** † Key Laboratory of Advanced Materials (MOE), Department of Chemical Engineering, Tsinghua University, Beijing 100084, China; ‡ Xinjiang Blue Ridge Tunhe Sci. & Tech. Co., Ltd. Changji 831199, Xinjiang, China

## Abstract

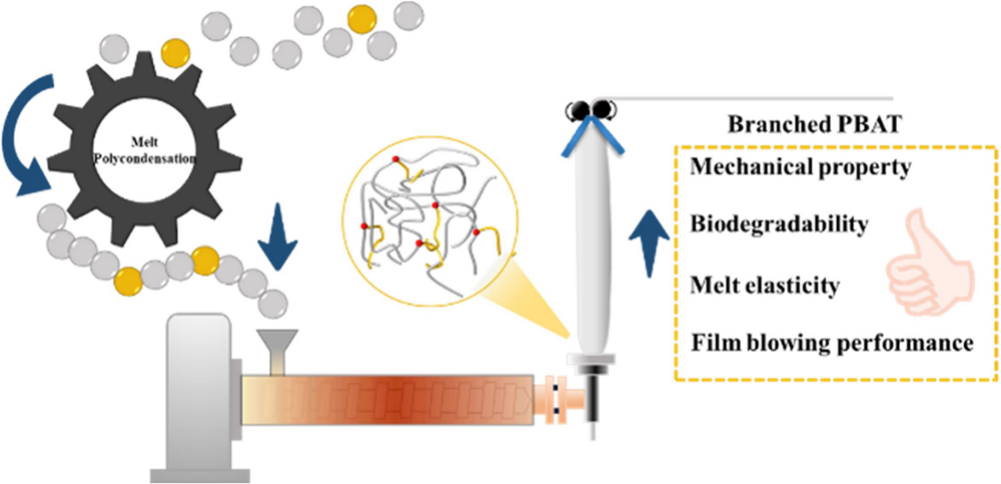

Poly(butylene adipate-*co*-terephthalate) (PBAT) is one of the most promising biodegradable copolyesters for addressing “white pollution” and has made significant progress in industrial production, particularly in packaging and mulching film applications. However, melt elasticity and the rapid biodegradation rate of linear PBAT limit the broader application. In this study, a series of novel PBAT copolyesters with a low content of branch units (1.3–1.6%) were synthesized from adipic acid (AA), terephthalic acid (PTA), 1,4-butanediol (1,4-BDO), and branching monomers with fixed side chain length through esterification and polycondensation. A comprehensive investigation into the macromolecular structure of branched PBAT was conducted. A “Memoryless Random Model” combined with ^1^H-NMR analysis was used to calculate the number-average sequence length, which proved the existence of random block sequences. Both linear and branched PBAT copolymers exhibited relatively high and similar molecular weights of 6.3 × 10^4^ Da and above. The conformational parameters *g* and *B*_3*w*_ were determined by gel permeation chromatography (GPC) coupled with triple detectors, thereby providing a clear definition of the branching characteristic. Fourier transform infrared spectroscopy (FTIR), differential scanning calorimetry (DSC), and X-ray diffraction (XRD) analyses revealed two crystalline forms in PBAT, with the β-form predominating in linear PBAT and a decrease in crystallinity in branched PBAT. The enzymatic hydrolysis experiment showed that the branched side chain reduced the hydrolysis rate, and the existence of consecutive sequences (≥3) was proved by LC–MS. The copolymers demonstrated higher complex viscosity and tanδ at the same frequency, indicating enhanced melt viscosity and elastic response. The deviation of the end slope in the Han curve also proved the existence of BA-BT random block sequence and complex relaxation behavior.

## Introduction

To address the issue of “white pollution” caused by nondegradable plastic products such as polyethylene (PE), polypropylene (PP), etc., the development of biodegradable materials as alternatives to common plastics presents a promising strategy for short-term uses, including packaging and agricultural applications like mulch films.^1−3^

At present, there are two main sources of biodegradable materials: one is biobased materials, such as starch, cellulose, chitosan, PHA, and PLA and the other is fossil-based materials such as PBAT, PPC, and PCL.^4−9^ Among these, poly(butylene adipate-*co*-terephthalate) (PBAT) stands out as a potential biodegradable polymer due to its balanced mechanical properties and thermal stability. Besides, its softness and transparency are comparable to low-density polyethylene (LDPE), making it suitable for processing into mulch films, packaging, and other film products.^10−12^

To enhance the properties of PBAT, various strategies such as blending,^13−15^ composite formation,^16^ and chain extension^17^ have been investigated to improve its mechanical and thermal performance. However, the highly linear chain structure and low molecular weight of PBAT result in insufficient melt elasticity and a rapid biodegradation rate, thereby limiting its broader application, particularly in film products.

Branched polymers offer significant advantages over their linear counterparts.^18^ By adjustment of the branch length and branching density, it is possible to create branched polymers with customized properties. The unique topological structure and performance characteristics of branched polymers have been widely studied, particularly in the context of polyolefins.^19−21^ Collectively, numerous studies have focused on the synthesis of branched polyesters through the incorporation of branching agents with diverse chemical structures.^22−26^ These agents include compounds containing multiple hydroxyl and epoxy functional groups, such as 1,1,1-tris(hydroxymethyl)ethane,^27^ tetrahydrophthalate (DGT),^28^ glycerol,^29^ epoxy-terminated branched polymer (ETBP),^30^ pentaerythritol (PER),^31^ and radical initiators like dicumyl peroxide (DCP).^32^ The investigation of branched PBAT has received attention, though studies focusing on well-defined branched polyesters remain limited. Incorporating additives with trifunctional or higher functionality often leads to the formation of cross-linked systems and an uncertain side chain length.

Such challenges impede a comprehensive understanding of branched PBAT structures. Therefore, investigating the architectural structure of well-defined branched PBAT is important, as it may illuminate the relationship between branching and material performance, paving the way for enhanced applications in sustainable materials.

**Scheme 1:**
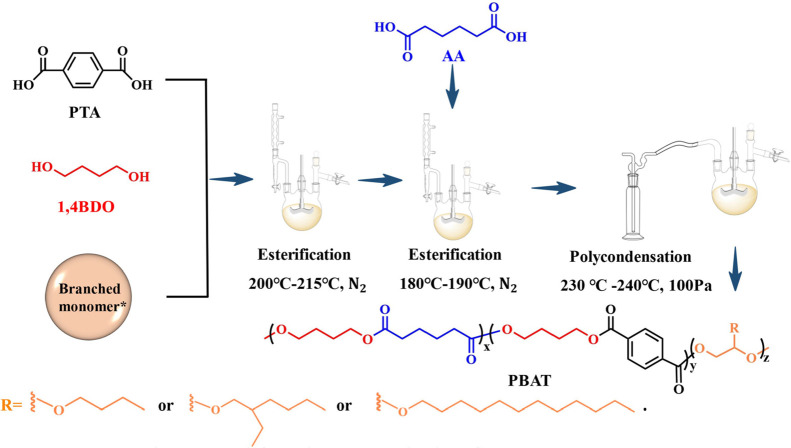
Synthesis Route for Linear and Branched PBAT Copolymers Molecular structure and physical property information are shown in Figure S1.

In this study, linear and branched poly(butylene adipate-*co*-terephthalate) (PBAT) copolyesters were synthesized from adipic acid (AA), terephthalic acid (PTA), 1,4-butanediol (1,4-BDO), and three branch monomers with fixed side chain lengths (Scheme 1). The synthesis was successfully conducted by using tetrabutyl titanate (TBT) as a catalyst through a two-step process of esterification and polycondensation. The effects of the branch units on the multilevel structure and enzymatic hydrolysis were investigated in detail. Furthermore, the degradation mechanisms influenced by the branched units were elucidated by using density functional theory (DFT) and molecular dynamics (MD) simulations, offering insights into the underlying processes. This study proposes an in-depth understanding of how the introduction of branched units influences the complex architectures in PBAT. It highlights the potential of the resulting branched PBAT for use in mulch films.

## Experimental Section

### Materials

PTA, AA (AR) were provided by Shanghai McLean Biochemical Technology Co., Ltd. 1,4-BDO (AR) was supplied by Shanghai Titan Technology Co., Ltd. Butyl glycidyl ether (bge), ethylhexyl glycidyl ether (ege), and glycidyl lauryl ether (gle) were provided by Shanghai Aladdin Biochemical Technology. Tetrabutyl titanate (TBT, 99.5%) as a catalyst was obtained from Zancheng (Tianjin Technology Co., Ltd.). All chemicals and solvents were used as received without further purification.

### Synthesis of Samples

Linear and branched PBAT copolymers were synthesized through a two-step esterification and polycondensation reaction in the melt state. In detail, PTA (18.7 g, 112.5 mmol), 1,4-butanediol (27 g, 300 mmol), and the branch monomer (6.25 mmol) were placed into a three-necked flask (250 mL) equipped with a condenser. The reaction was carried out at 215 °C with a mechanical stirrer under nitrogen protection for 3 h. Then, AA (20 g, 134.5 mmol) and TBT (30 μL) were added to the reactor. The temperature was increased slowly to 180 °C, and the heating was continued with a mechanical stirrer under nitrogen protection for 3 h. The end of the esterification process was when 90% of the theoretical water yield was collected in the water separator. In the following polycondensation process, the flask was connected to a vacuum pump through a cold trap and heated to 230 °C at a pressure below 100 Pa for 3 h. The hot copolymer melt was transferred to a polyimide film, cooled, and analyzed. The synthesized polyesters were denoted as PBAT, PBAT-bge, PBAT-ege, and PBAT-gle, respectively.

### Characterization

The molecular weights (*M*_n_ and *M*_w_) of PBAT polyesters were measured at 35 °C with gel permeation chromatography (Waters 2414) with three detectors (Waters 2414, Wyatt Treos II and Wyatt ViscoStar III). Chloroform was used as a solvent, and the flow rate of the eluent was 1 mL/min.

The chemical compositions of PBAT samples were confirmed by ^1^H NMR spectroscopy, which was recorded on a JNM-ECA600 NMR spectrometer with deuterated chloroform as the solvent. FT-IR spectroscopy was recorded on a Thermo Scientific-Nicolet 6700 with reflection mode, wavenumber ranging from 4000−500 cm^–1^.

Tensile properties were measured at 25 °C and a crosshead speed of 50 mm/min with a Jinjian UTM-1432. Dumbbell-shaped specimens of 25 mm length, 4 mm width, and 2 mm thickness were made by hot-pressing at 160 °C and 30 MPa for 2 min. Each measurement was repeated at least five times, and the values were averaged.

Differential scanning calorimetry (DSC) analysis was carried out under a N_2_ atmosphere to study the thermal behavior of samples using TA-DSC 250 equipment. The samples were first heated from 30−160 °C with 10 °C/min ramp and held at 160 °C for 3 min to eliminate thermal history, then subsequently cooled to −50 °C with 10 °C/min ramp and held at −50 °C for 3 min. Finally, the second heating run was performed by heating the samples to 160 °C at a rate of 10 °C/min.

The thermal decomposition behavior was measured with a TGA (Shimadzu DTG-60). The samples were heated from room temperature to 500 °C at 10 °C/min under N_2_ atmosphere.

Wide-angle X-ray diffraction analysis was carried out at room temperature by using a WAXD-Bruker D8 Advance diffractometer with Cu–Kα radiation. Scanning was performed with 2θ from 5° to 35° at a rate of 4 °/min with a step of 0.02°.

The rheology tests were conducted on an Anton Paar Physica MCR301, with a parallel plate geometry of 8 mm diameter. The plate gap was about 1 mm in each test, and the temperature was 160 °C. A dynamic frequency sweep analysis was performed at a strain of 1% with a frequency from 100 to 0.1 rad/s.

Enzymatic degradation tests were performed in a water bath shaker at 37 °C. The phosphate buffer containing cutinase was used in the experimental group, and 8 mL of phosphate buffer was used in the control group. The films were washed and dried to a fixed weight at regular intervals. The degradation level was estimated from the percentage of weight loss according to the following relationship:
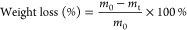
1where *m*_0_ is the original weight, and *m*_t_ is the weight after degradation.

SEM (Jeol 7900f) was carried out to observe morphology changes during degradation at an accelerating voltage of 20 kV. All samples were coated with a layer of platinum.

## Results and Discussion

### Chain Structure

**Figure 1:**
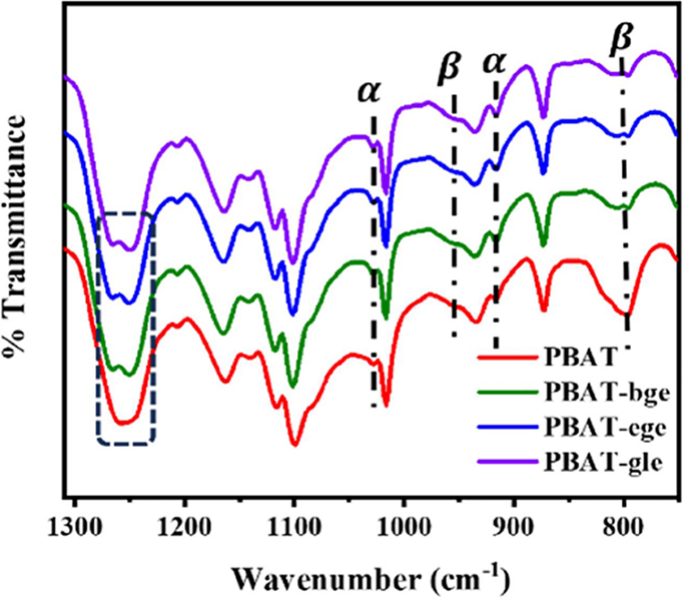
FTIR spectra of the linear and branched PBAT copolymers at 1310–850 cm^–1^. (The terms “α” and “β” in Figure 1 are assigned two different crystal forms in PBAT which are discussed in “crystalline structure”).

The chain structure of the branched and linear PBAT copolymers was confirmed by FTIR and ^1^HNMR. In the infrared (IR) spectrum of PBAT (Figure 1), the absorption peaks at 1310–850 cm^–1^ are typically associated with specific molecular group vibrations and crystalline structures. These absorption peaks are of significant importance for understanding the chemical structure and molecular characteristics of PBAT.

The C–O bond forms part of the ester group. This bond exhibits strong absorption, typically manifesting as prominent peaks within the 1240–1260 cm^–1^ range. Additionally, the deformation vibrations of −O–CH_2_– and −C–CH_3_– groups within the branch chain may also contribute to absorption in this region. In branched PBAT, the incorporation of branching units introduces more long-chain −O–CH_2_– and -C–CH_3_ groups into the molecular structure. This results in distinct peak profiles in the infrared spectrum when compared to linear PBAT, highlighting how the deformation vibrations of these alkoxy and long-chain −C–CH_3_ groups alter the molecular structure of linear PBAT. The other functional group analysis is shown in Figure S2.

**Figure 2:**
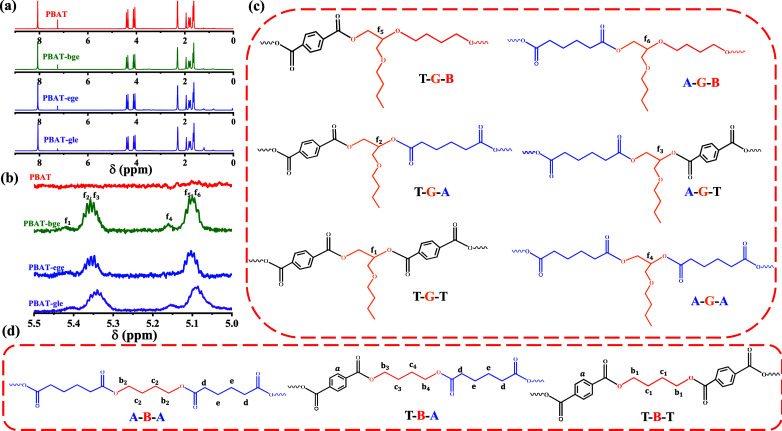
(a) ^1^H NMR spectra of linear and branched PBAT copolymers, (b) enlargement of chemical shifts at 5.0–5.5 ppm, (c) chain structures of the branched units of PBAT copolymers, and (d) chain structures of the linear units of PBAT copolymers.

To gain deeper insight into the chain structures of the PBAT copolymers, ^1^H NMR analysis was performed for all samples. As shown in Figure 2, the linear PBAT segment units can adopt three possible sequences: TBT, ABT, and ABA, while the branched PBAT segment units can adopt six possible sequences: TGB, AGB, AGA, TGA, TGT, and AGT.

The different connection sequences in the PBAT segment units resulted in varying chemical shifts on the ^1^H NMR spectrum due to the distinct chemical environments. Among these, the (Ha) signal of the benzene ring appeared at 8.0–8.1 ppm. Considering the inductive and conjugative effects on electron cloud density, the hydrogen protons adjacent to the ester group on butanediol exhibited chemical shifts at 4.4 ppm (Hb1) from the TBT segment, 4.3 ppm (Hb3) and 4.1 ppm (Hb4) from the TBA segment, and 4.0 ppm (Hb2) from the ABA segment. Considering that the negative effects are weakened along the carbon chain, the (Hc) peaks appeared at a lower chemical shift than the adjacent peaks (Hb). It can be analyzed that 2.0 ppm is the chemical shift of butanediol (Hc1) on the TBT segment, 1.7 ppm is the chemical shift of butanediol (Hc2) on the ABA segment, and 1.9 and 1.8 ppm are the chemical shifts of butanediol (Hc3) and (Hc4) on the ABT segment, respectively. Additionally, the signals at 2.3 and 1.6 ppm were attributed to the adipic acid protons (Hd) and (He).

Compared with linear PBAT, the ^1^H NMR spectra of three branched PBAT copolyesters exhibited small but significant characteristic peaks at 5.1–5.4 ppm (Hf1–6). It can be determined that these characteristic peaks belong to the chemical shift C–H in the branch units. Considering the six branched structure sequences formed by the ring-opening reaction of the epoxy functional group of the branched units. The peaks at 5.35 ppm (Hf2, Hf3) and 5.08 ppm (Hf5, Hf6) are assigned to the CH protons between the mixed sequences of T–G–A, A–G–T and T–G–B, A–G–B, respectively.

To ensure that the copolyesters exhibit both good mechanical properties and biodegradability, the feed ratio of PTA and AA is controlled at around 45:55. The molar percentages of PTA (*n*_PTA_), AA (*n*_AA_), and branched monomer (*n*_br_) were calculated using eqs 2–4.
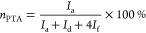
2
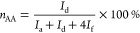
3
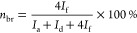
4

Furthermore, the number-average sequence lengths of BA (*L*_BA_), BT (*L*_BT_) were calculated using eqs 5 and 6. Randomness (*R*) were calculated using eq 7. The *R* values can reflect the randomness of the connection. In this study, *R* values close to 1 indicated that all of the copolymers had a random sequence structure.
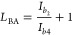
5
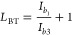
6
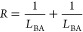
7

To further investigate the impact of branching structures on the sequence of molecular chains in branched PBAT, a transfer matrix was introduced to analyze the distribution of molecular segments in the random copolymers. Based on the initial feed ratio, the initial state probabilities were set, and a discrete “Memoryless Random Model” was employed to simulate the distribution of molecular chain segment sequences. Considering that the actual products had similar molecular weights, the chain length was set to 1000. The description of the transfer matrix “Memoryless Random Model” can be seen in the Supporting Information.
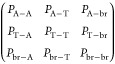
8

In the molecular chain, a continuous distribution of block sequences was observed with a major fraction (≥95%) of block lengths of less than 3. The introduction of branched units particularly led to an increased probability of consecutive blocks of more than 4 (Figure S3). Additionally, we compared the number average sequence lengths derived from our model with those obtained from ^1^H-NMR, revealing a strong correlation that confirms the accuracy of the model. The copolymer composition, average sequence length in the chain are summarized in Table 1. Detailed information on ^1^H-NMR spectra was provided in Figure S4.

**Table 1:** Molecular Short-Range Structures of Linear and Branched PBAT Copolymers

sample	*n*_AA_^a^	*n*_PTA_^a^	*n*_br_^a^	*n*_AA_^b^	*n*_PTA_^b^	*n*_br_^b^	*L*_BA_	*L*_BT_	*L*_BA_^c^	*L*_BT_^c^	*R*
PBAT	55.0	45.0	0	54.6	45.4	n.d	2.03	1.83	1.87	1.62	1.04
PBAT-bge	54.0	44.0	2.0	55.3	43.4	1.3	2.13	1.95	1.88	1.60	0.97
PBAT-ege	54.0	44.0	2.0	52.8	45.8	1.4	2.08	1.90	1.83	1.59	1.01
PBAT-gle	54.0	44.0	2.0	53.1	45.3	1.6	2.03	1.89	1.79	1.63	1.01

a The molar percentage of AA (*n*_AA_), PTA (*n*_PTA_), and branched monomer (*n*_br_) in feed ratio.

b The molar percentage of AA (*n*_AA_), PTA (*n*_PTA_), and branched monomer (*n*_br_) calculated from ^1^H NMR spectra.

c The number-average sequence length of BA (*L*_BA_), BT (*L*_BT_) was obtained by.

### Determination of Branching Characteristics

The chain structure is a short-range structure of polymers. Further in-depth study of the size and morphology of the molecular chain in dilute solution was carried out to clarify the unique structural parameters of branched PBAT. Through the control of the polymerization process, the branched PBAT-bge, PBAT-ege, and PBAT-gle had *M*_w_ values of 67–70 kDa, with PDI ranging from 1.37 to 1.40, which was similar to that of linear PBAT (As seen in Figure S5).

The mean square radius of gyration < *R*_g_^2^> is a parameter of the chain conformation of the polymer in dilute solution measured by SEC-MALS. To gain a deeper understanding of the structural differences between branched and linear PBAT molecules, the contraction factor *g* was introduced. This factor quantified the dimensions of branched polymers by representing the size ratio, specifically the ratio of the mean square radius of gyration of branched molecules to that of linear molecules with the same molecular weight, as noted in eq 9.
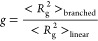
9

**Figure 3:**
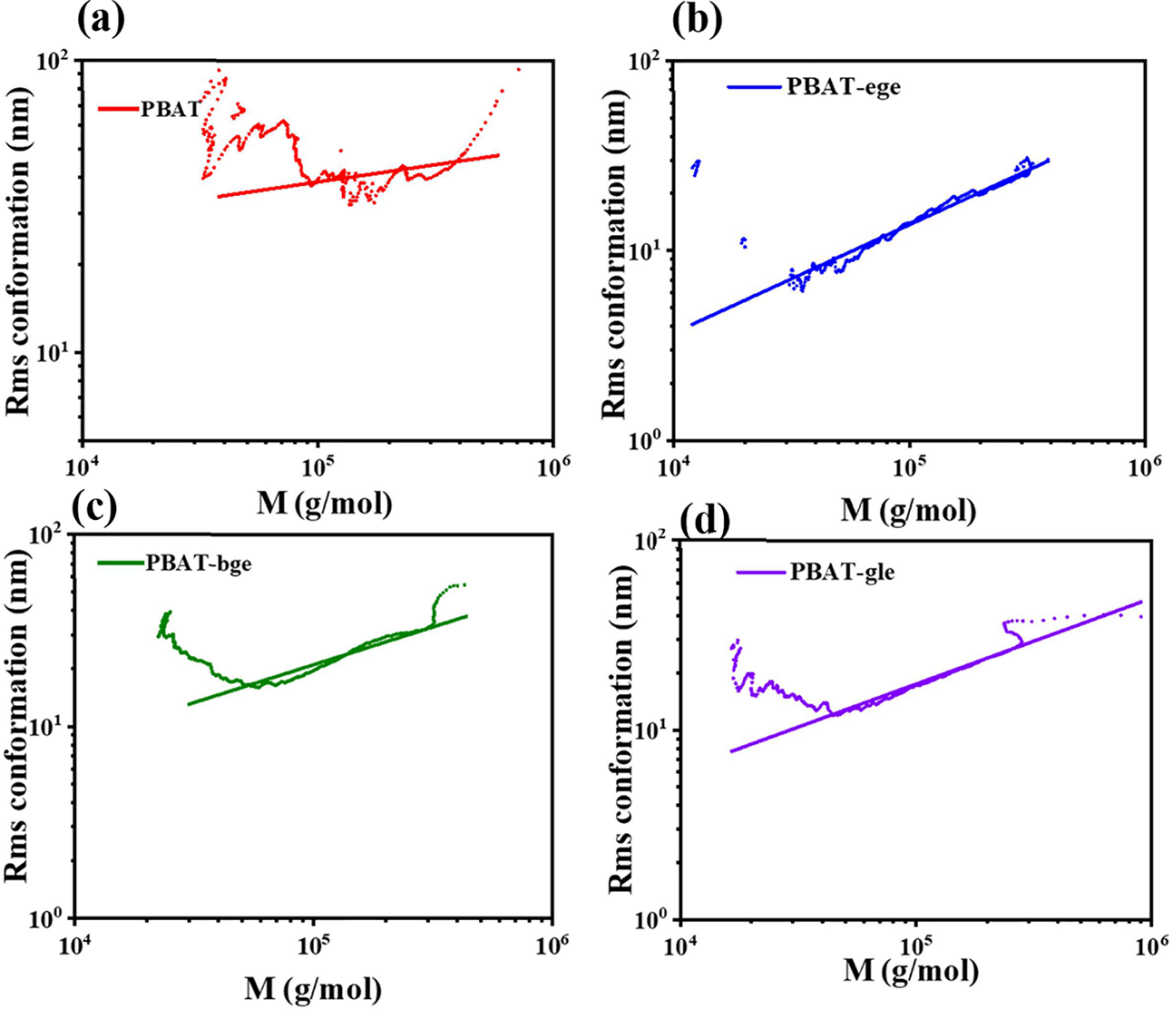
Rms conformation plot and linear fit of linear and branched PBAT copolymers: (a) linear PBAT, (b) PBAT-ege, (c) PBAT-bge, and (d) PBAT-gle.

The *R*_g_ value of linear PBAT was 25.7 nm through computer iterative fitting. The similar molecular weight of the branched PBAT shows average *R*_g_ values between 16.3 and 21.0 nm. In the linear region shown in Figure 3, it can be observed that the branched PBAT had a smaller hydrodynamic volume, which indicated that under the same solvent environment and molecular weight, the branched PBAT showed a more compact random coil. Due to the limitations of the characteristics of the light scattering instrument, it has low sensitivity for shorter molecular chains. The lower size limit of light scattering (*R*_g_ ≈ 10 *nm*), below which particles act as isotropic scatterers, potentially “cutting off” the low-molar-mass tail that is present in Figure 3.

Zimm and Stockmayer had made a detailed theoretical derivation of the contraction factor *g* of branched polymers, specifically for random copolymer molecules containing only trifunctional branching units.^33^
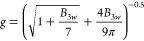
10

Given that this is an expression of monodisperse or narrow distribution. For polydisperse randomly branched molecules with trifunctional branch points, the expression of contraction factor *g* will become as eq 11.^34^
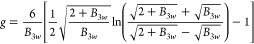
11

*B*_3*w*_ is the average number of branch points per molecule and is a function of the molecular weight *M*, which can be constructed through eq 12.^35^
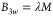
12

The parameter λ defined as the branching frequency can be calculated from eq 13.
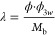
13

ϕ_3*w*_ is the ratio of the mass of the branching molecule to the mass of the branched polymer (in g/g polymer), and *M*_b_ is the molecular weight of the branching molecule. ϕ can be interpreted as the number of effective branches without consideration of the linear backbone of the chain, so ϕ values range from 0 to 1. One can calculate ϕ via the average effective functionality *f̅* ,which is the average number of chains starting from a branch point and including the backbone. For trifunctional branching molecules:
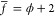
14

It was worth noting that the branch unit we added in this work seemed to be a reactive monomer with only two functions; however, it can be considered as a trifunctional branching unit reactive monomer with three functional degrees. The epoxy functional group has two reactive active functional degrees, and one reactive active site is connected to a branched chain of fixed length. Namely, all branch points in the actual molecular chain composition must have a branch chain introduced, so the ϕ = 1. And the expected result was *f̅* = 3 for branched PBAT. We compared the theoretical *B*_3*w*_ with the experimentally measured *B*_3*w*_ and found close agreement. This data demonstrated that each polymer chain typically had 3–4 branching sites.

The viscosity data were used to generate Mark–Houwink–Sakurada (MHS) plots that relate the intrinsic viscosity of a polymer ([η]) to the molar mass (M) in a logarithmic coordinate system through eq 15.
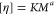
15

**Figure 4:**
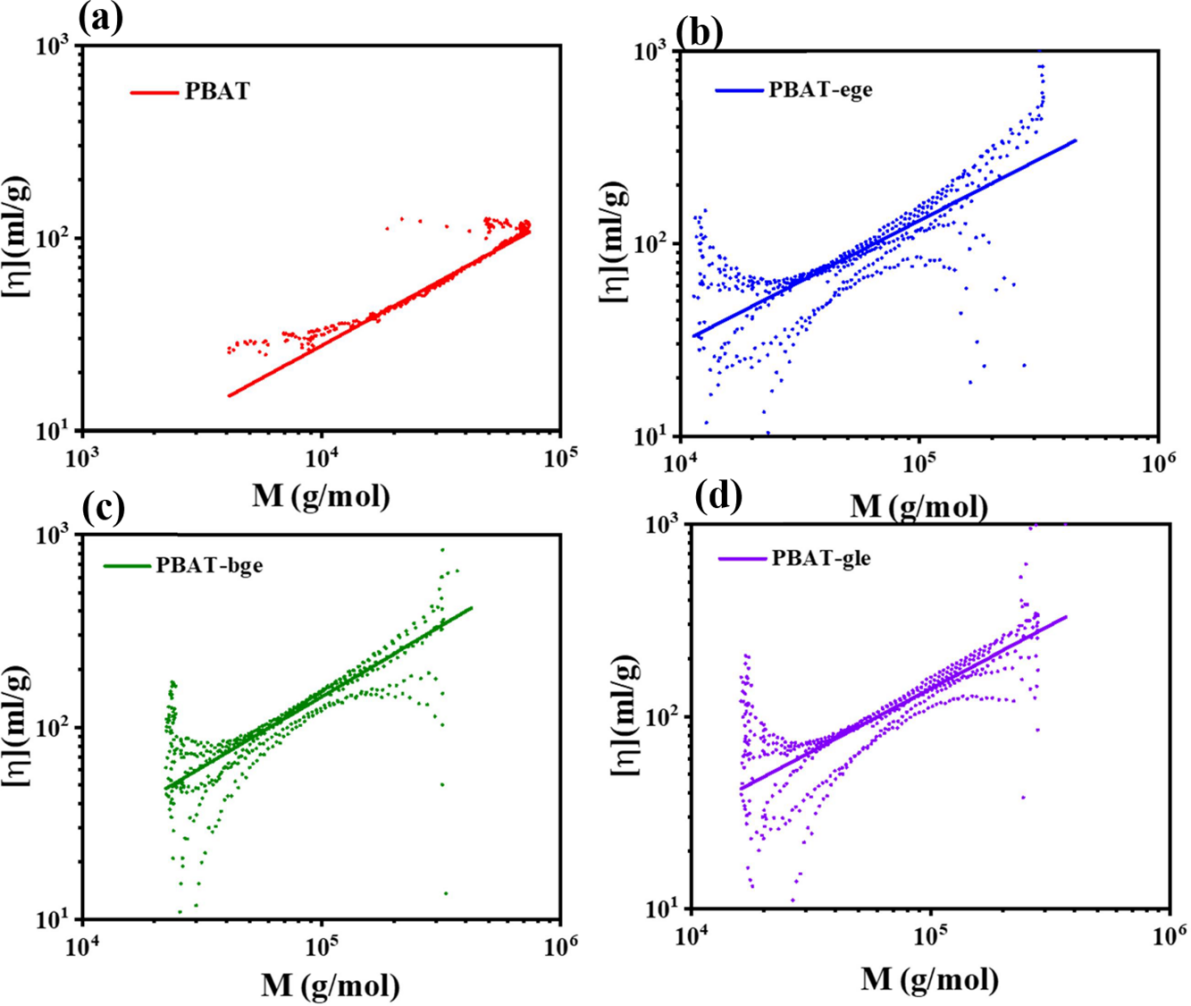
Intrinsic viscosity plot and linear fit of linear and branched PBAT copolymers: (a) linear PBAT, (b) PBAT-ege, (c) PBAT-bge, and (d) PBAT-gle.

As seen in Figure 4, the conformation parameter a of branched PBAT ranged from 0.671 to 0.718, which was smaller than 0.731 for linear PBAT. This indicated that the presence of low content branching units could reduce the hydrodynamic volume of the PBAT random coil in the solvent, leading to a higher packing density. The contraction factor *g′* can also be calculated by taking the ratio of the intrinsic viscosity of the branched polymer [η]_branched_ to that of its linear counterpart [η]_linear_ for each molar mass fraction.
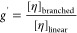
16

Given the significant error associated with the *R*_g_ obtained from multiangle laser detectors, *g*′ was considered more reliable than *g*. However, both factors were crucial for gaining insights into the conformation of branched polymers, and extensive research has been dedicated to exploring this relationship. Extensive research indicates the exponential relationship shown in eq 17 between *g′* and *g*.
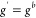
17

The value of exponent *b* depends on the properties of a polymer coil. It was first suggested that *b* = 1.5 by Zimm and Stockmayer.^33^ Zimm and Kilb^36^ showed that the exponent has the values of 0.5 for a nondraining polymer and 1.0 for a free-draining polymer. Experimental results for branched LDPE and branched PS have generally supported the use of the exponent 0.5.^37−39^ All theories were valid only for theta solvents, so that modifications were necessary for good solvents in which GPC measurements usually were carried out. In this study, we determined *b* values between 0.63 and 0.74 for randomly branched PBAT, which represents new progress that has been made in the research of branched polyester.

All the parameters included molecular weight, intrinsic viscosity [η], root-mean-square radius *R*_g_, and constants with branching characteristics, Mark–Houwink-Sakuruda constants *K* and *a*, branching frequency are summarized in Table 2.

**Table 2:** Molecular Long-Range Structures of Linear and Branched PBAT Copolymers

sample	*M*_w_ (kDa)	*M*_n_ (kDa)	DPI	*R*_g_ (nm)	ϕ_3*w*_	*B*_3*w*_^a^	*B*_3*w*_^b^	[η] (mL/g)	*K*	*a*	*b*
PBAT	71.2	56.1	1.27	25.7	n.d	n.d	n.d	101	0.036	0.731	n.d.
PBAT-bge	70.1	51.4	1.37	19.0	1.23	3.91	3.41	89	0.034	0.718	0.74
PBAT-ege	67.5	48.6	1.39	17.5	1.76	3.76	3.32	85	0.033	0.716	0.70
PBAT-gle	70.7	50.8	1.40	16.3	2.29	4.79	4.05	90	0.051	0.671	0.68

a The average number of branch points per molecule in Zimm and Stockmayer theory.

b The average number of branch points per molecule derived from GPC test.

### Crystalline Structure

**Figure 5:**
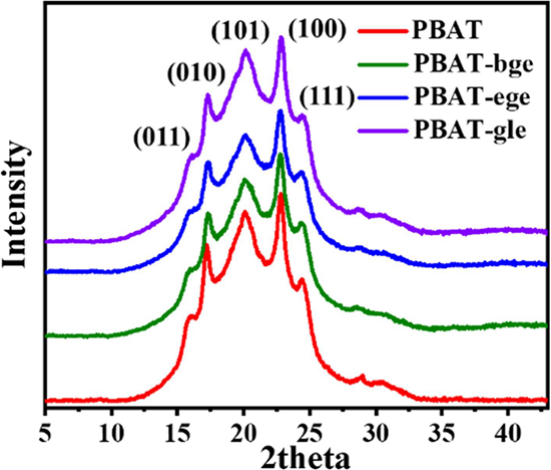
XRD patterns of linear and branched PBAT samples.

To further understand the influence of branch units on the crystallization structure of the copolyesters, XRD analysis was conducted for all of the PBAT copolymers. As shown in Figure 5, all of the PBAT copolymers exhibited five main peaks at around 2θ = 16.1°, 17.3°, 20.1°, 22.8°, and 24.5°, corresponding to the (011), (010), (101), (100), and (111) reflection planes of PBT-like crystals. No peaks corresponding to the crystal structure of PBA were observed in this study, indicating that only PBT crystals were present in PBAT.^40^

The X-ray diffraction (XRD) patterns of PBAT revealed distinct diffraction peaks at 31.5° for the (104) plane of the α-form of polybutylene terephthalate PBT and at 28.3° for the (104) plane of the β-form of PBT.^41^ Due to the relatively weak intensities of these diffraction peaks in the wide-angle X-ray diffraction (WAXD) profiles, further investigation of the crystalline structure in both branched and linear PBAT samples was carried out using FTIR spectroscopy, as shown in Figure 1.

The FTIR spectra of branched and linear PBAT showed characteristic absorptions at 916, 1024, 954, and 830 cm^–1^. These absorption features closely resembled those of the α and β crystals of neat PBT.^41^ The results indicated that both linear and branched PBAT samples contained a mixture of the two crystalline forms. However, the introduction of branched chain structures in the branched PBAT led to a higher proportion of the α crystalline phase, whereas the linear PBAT exhibited a predominance of the β crystalline phase. This observation aligned with the findings from XRD analysis.

The presence of branched chains particularly favored the formation of more α forms during cooling crystallization. However, the crystallization behavior was highly dependent on the number and distribution of these branched chains. While they facilitated nucleation, they also disrupted the orderly arrangement of the polymer backbone, ultimately reducing the overall crystallinity of the copolyester. The crystallinity (*X*_c_) was calculated through peak splitting fitting, showing a decrease from 20.83% for PBAT to 14.36% for PBAT-ege.

**Figure 6:**
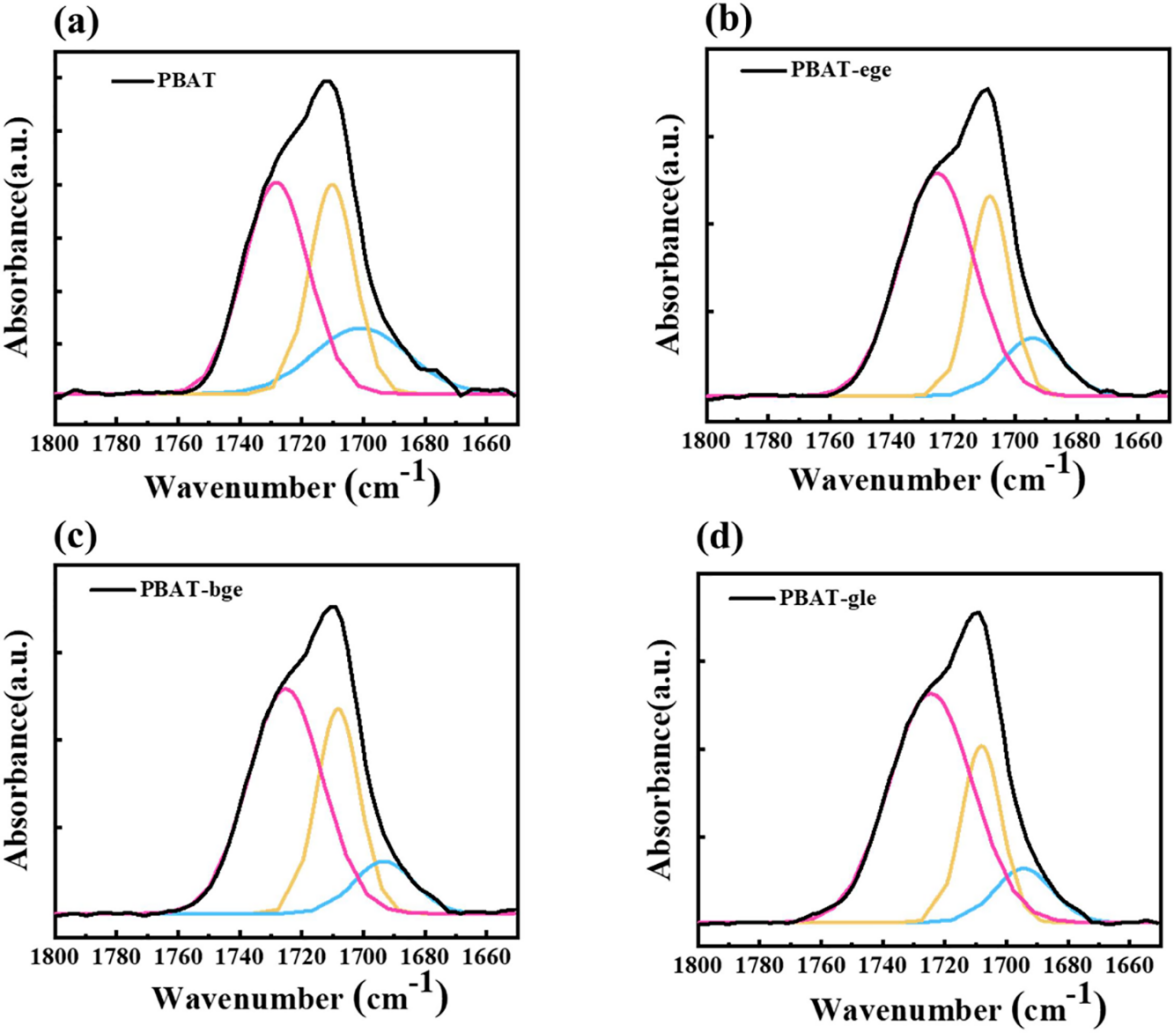
FTIR spectra of the linear and branched PBAT copolymers at 1650–1800 cm^–1^: (a) linear PBAT, (b) PBAT-ege, (c) PBAT-bge, and (d) PBAT-gle.

The morphology of the infrared absorption peaks corresponding to the C=O groups was believed to reflect the distribution of crystalline and amorphous regions within the condensed structure.^42−44^ By fitting the infrared absorption peaks of the carboxyl group within the range of 1650–1800 cm^–1^, three distinct absorption bands at 1728, 1710, and 1700 cm^–1^ were identified in linear and branched PBAT (Figure 6). These bands were associated with the amorphous, intermediate, and crystalline phases of the C=O stretching vibration absorption peak in polyester, respectively.^45,46^ Normalization of the relative proportions of different peak areas indicated that the introduction of branched units increased the amorphous fraction of PBAT, consequently reducing its crystallinity (Table S3).

The absorption peak position of branched PBAT showed a wavenumber reduction of approximately 3–6 cm^–1^. This minor shift was primarily attributed to variations in the packing density within the crystalline regions and the distinct conformations of the carbonyl groups in the amorphous phase. The incorporation of branches introduced a significant number of −CH_3_ groups, which participated in C–H···O interactions, recognized as a form of weak hydrogen bonding. These weak hydrogen bonds induced subtle shifts in the carbonyl absorption peak position, aligning with predictions from quantum chemical calculations by Dybal et al.,^47^ demonstrating good agreement between theoretical and experimental data.

### Thermal Property

**Figure 7:**
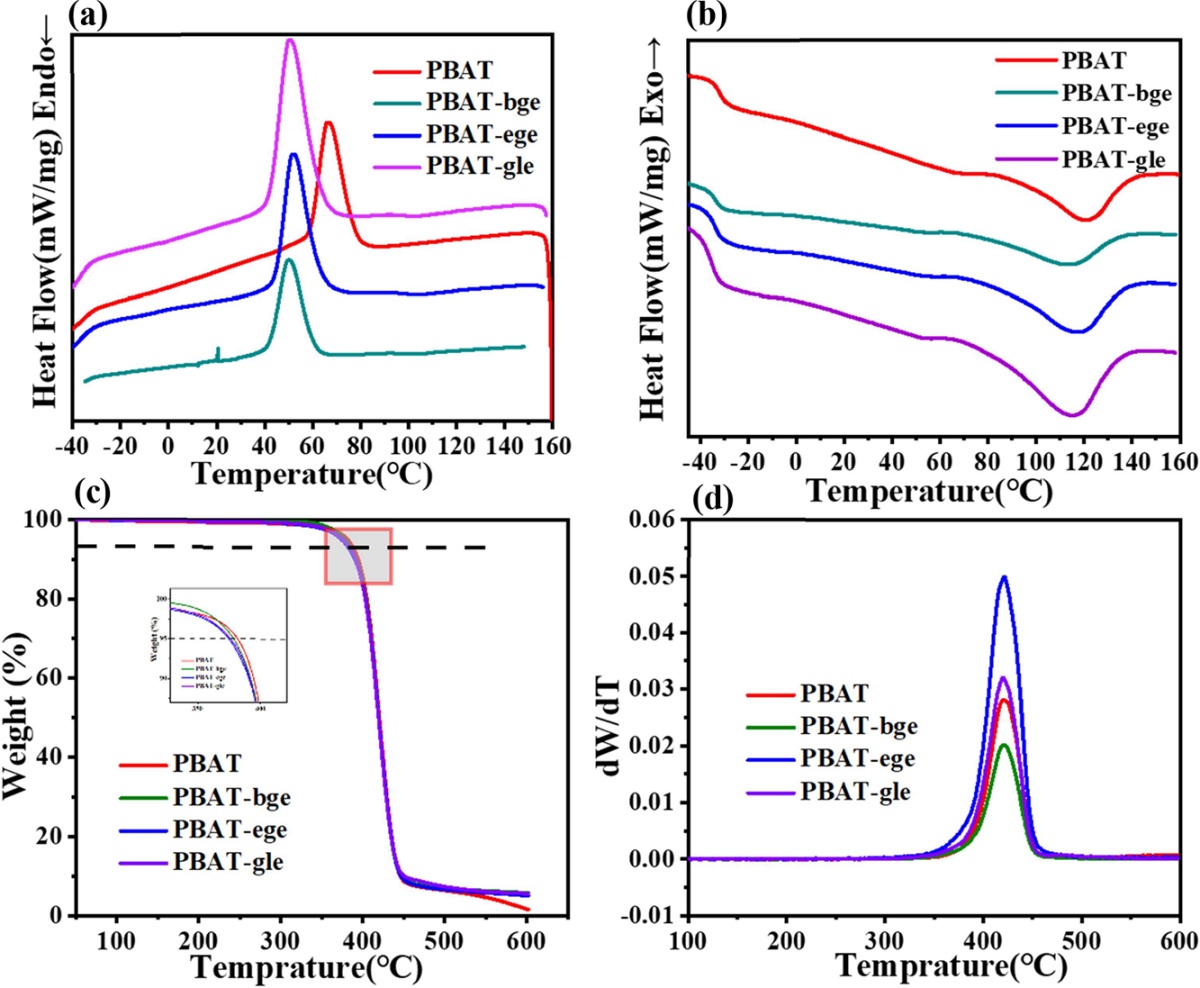
DSC curves of (a) cooling, and (b) second heating of linear and branched PBAT samples; TGA (c) and DTG (d) curves of linear and branched PBAT samples under N_2_ atmosphere.

Differential scanning calorimetry (DSC) was used to measure the thermal properties of linear and branched PBAT copolyesters. The glass transition temperature (*T*_g_), melting temperature (*T*_m_), and crystallization temperature (*T*_c_) of linear and branched PBAT were investigated and summarized in Table 3. As shown in Figure 7a,b, the *T*_g_ of branched PBAT systematically decreased with similar BA to BT weight fractions of linear PBAT, from −32.0 to −35.3 °C. This decrease was attributed to the increased free volume of the main chain, as confirmed by studies on other branched aliphatic and aromatic polyesters.^48−50^

**Table 3:** Thermal Properties of Linear and Branched PBAT Samples

sample	Δ*H*_c_ (J/g)	*T*_c_ (°C)	*T*_g_ (°C)	*T*_m_ (°C)	Δ*H*_m_ (J/g)	*T*_5%_ (°C)	*X*_c_ (%)
PBAT	19.0	66.2	–32.0	120.4	14.2	382	20.83
PBAT-bge	14.9	50.0	–33.4	113.3	10.4	376	15.42
PBAT-ege	21.5	51.8	–33.6	116.4	14.6	372	14.36
PBAT-gle	30.6	50.5	–35.3	115.1	19.1	374	14.78

The branched side chains in PBAT significantly influenced both crystallinity and the crystalline melting behavior. The introduction of branched units at a BA to BT ratio that is similar shifts the *T*_c_ to lower temperatures. This effect was attributed to the branched chains hindering the regular arrangement of molecular chains, with the short-branched chains unable to be incorporated into the lattice, creating defect points that increased the crystallization energy barrier.

As the length of the side chain increased, *T*_m_ decreased. This was because longer side chains offered greater flexibility, which led to a depression in *T*_m_.^51^ However, weak side chain crystallinity was observed in the three-branched PBAT, as evidenced by the increasing *T*_m_ of the three-branched PBAT.^52^ A slight melting transition appeared around 63 °C, corresponding to the melting of the PBAT-β crystal, which was prominently observed in linear PBAT.^53^ This further supported the observation from XRD and infrared spectroscopy that linear PBAT contained a higher proportion of the β crystalline phase.

The effect of adding branched units on the thermal stability of PBAT was characterized by thermogravimetric analysis (TGA). The thermal decomposition curves of PBAT with different structures were largely similar. However, there was a slight difference in the initial thermal decomposition temperature *T*_5%_. With the introduction of branched units, the *T*_5%_ of PBAT copolyesters decreased from 382 to 372 °C, likely due to the presence of unstable tertiary carbon atoms. The introduction of branched points resulted in a higher proportion of tertiary carbon atoms in the main chain. Hydrogen atoms attached to tertiary carbons are more susceptible to thermal oxidation compared to those on secondary and primary carbon atoms, leading to a lower thermal decomposition temperature for branched PBAT. Among the three types of branched PBAT, PBAT-bge contained the highest content of tertiary carbon atoms, resulting in the lowest thermal degradation temperature and the highest thermal degradation rate.

### Enzymatic Hydrolysis

The initial stage of biodegradation is hydrolysis, during which macromolecular polymers are degraded into smaller oligomers under the catalysis of enzymes. The concentration of cutinase was determined to be 0.6 mg/mL, as measured by the microplate reader method (Figure S6). Degradation was induced by the cleavage of ester linkages, leading to the formation of water-soluble oligomeric or monomeric products, which ultimately caused weight loss. The degradation products were identified by LC/MS during PBAT biodegradation, with the hydrolysis products primarily consisting of A, B, T, BTBA, BTBT, BABA, TBTBT, and other detected oligomers (Figure S7).

In the chain structure calculations, the average sequence lengths of BT and BA were established. The presence of trace amounts of longer block sequences observed in the degradation products supports our hypothesis regarding the formation of complex macromolecular sequence blocks.

**Figure 8:**
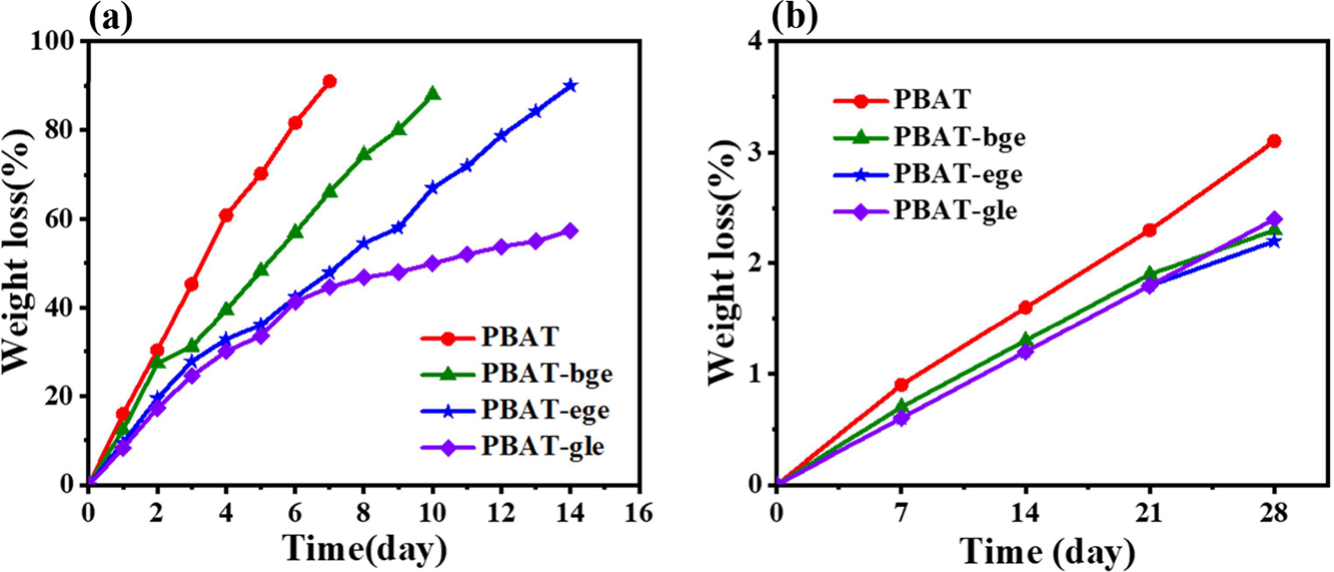
Degradation of linear and branched PBAT (a) cutinase solution at 37 °C; (b) phosphate buffer solution at 37 °C.

Figure 8a illustrates the in vitro enzymatic hydrolysis of PBAT. Samples in the control group (phosphate buffer solution at 37 °C) showed barely any degradation by the end of the test, with all samples exhibiting a weight loss of less than 5% (Figure 8b). The hydrolysis rate of branched PBAT was lower than that of linear PBAT, primarily due to the steric hindrance effects of the branched chains, which obstructed the recognition and binding of cutinase’s active pocket to the ester linkages of PBAT. Additionally, the presence of branched side chains increased hydrophobicity, reducing the affinity between the polyester surface and water molecules, thereby further inhibiting the hydrolysis process (Figure S8).

The degradation behavior was further studied using SEM (Figure S9), which revealed the microscale morphologies of the film surfaces. Initially, all sample surfaces appeared to be smooth. During the 15 days of enzymatic hydrolysis, more irregular holes emerged, although the holes were less pronounced with the addition of branched units. As the degradation time increased, fragments were removed from the surface, leading to wider cracks and more cavities within the films.

DFT and MD simulations were employed to investigate the mechanism by which branch units influenced PBAT enzymatic hydrolysis. The hydrolysis process of PBAT involves two key steps: first, the physical transport of water molecules to hydrolytically active sites to form complexes; second, the chemical hydrolysis reaction of the complex. Since the branch units were chemically inert in the degradation environment, they did not alter the chemical hydrolysis reaction but instead affected the physical transport of water molecules.

Two key aspects need to be considered in the physical transport process: the permeability of undissolved small molecules across the potential barrier into the phase region and the diffusion rate of the dissolved small molecules. DFT was employed to study the permeability of water molecules in both linear and branched PBAT, while MD simulations were used to examine the diffusion coefficient of dissolved water.

To investigate the diffusion behavior of water molecules, random copolymerization models of linear PBAT and branched PBAT (referred to as PBAT-0 and PBAT-graft, respectively) were constructed. The specific chemical composition of these models was presented in Figure S10.

**Figure 9:**
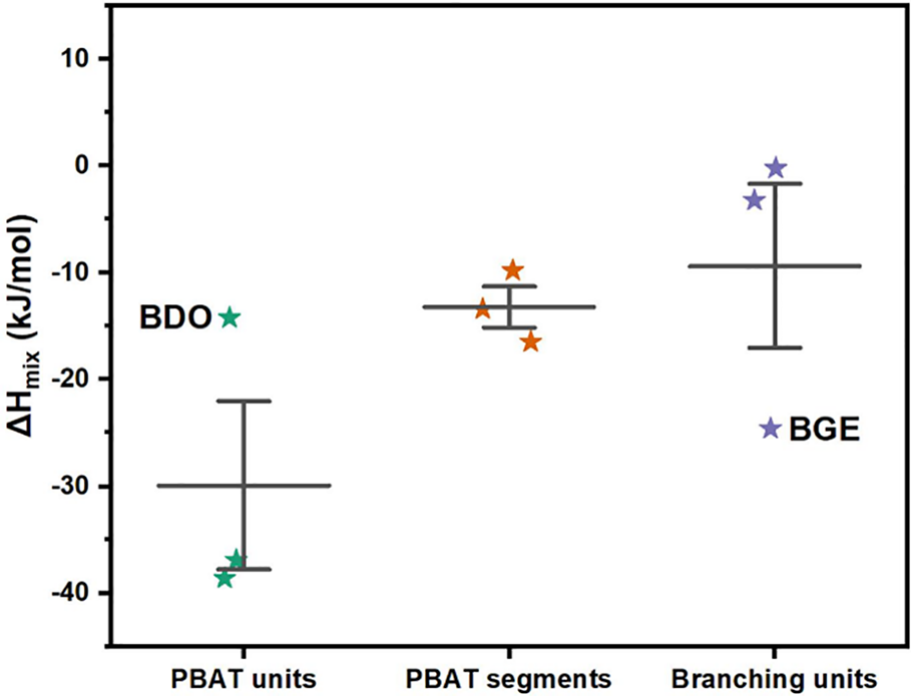
Δ*H*_mix_ of PBAT units, PBAT segments, and branching units.

Branch units inhibited the permeation process of the water molecules. As shown in Figure 9, the hydrophilicity sequence was PBAT units > PBAT segments > branching units, according to the Δ*H*_mix_. When branch units were introduced, the hydrophilicity of the PBAT chain decreased, and the branched chains hindered the penetration process of water molecules, reducing the probability of the formation of complexes between water molecules and the hydrolysis active center sites of PBAT.

**Figure 10:**
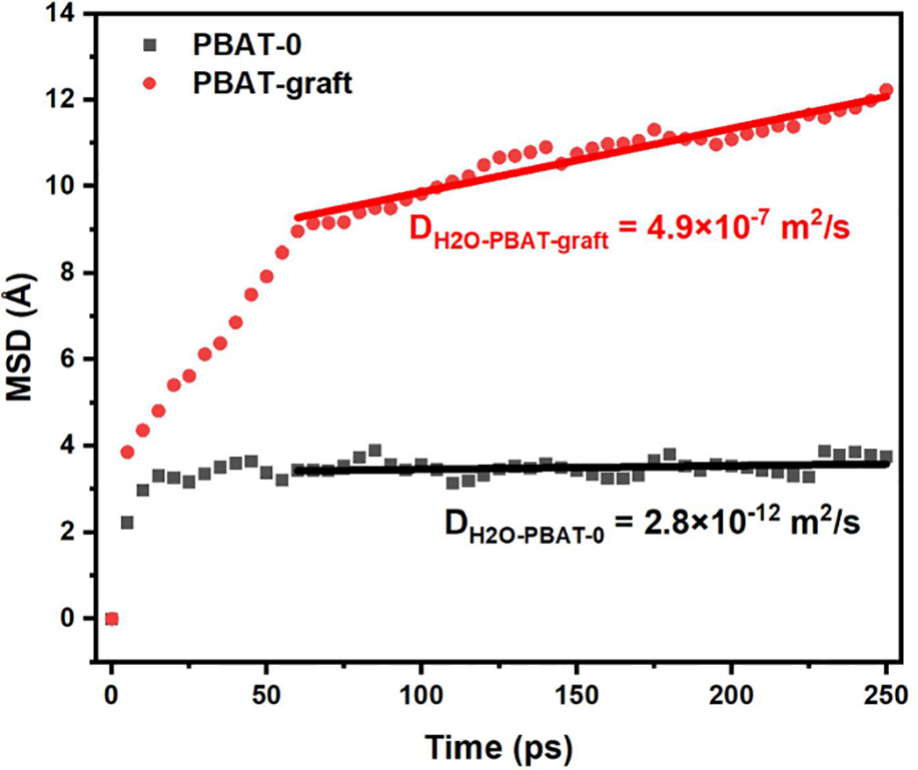
MSD time curves of water molecules in PBAT-0 and PBAT-graft models.

Branch units did not reduce the diffusion rate of the water molecules. As shown in Figure 10, the diffusion behavior of water molecules in PBAT-0 at room temperature was confined to the local pores of PBAT. In contrast, the mean square displacement (MSD) of water molecules in PBAT-graft exhibited an increasing trend over time. Throughout the entire time range, the diffusion rate of water molecules in PBAT-0 was slower than in PBAT-graft, with the diffusion coefficients following the relationship D(PBAT-0) < D(PBAT-graft). This was because the introduction of branch units increased the surrounding free volume, thereby facilitating the movement of nearby water molecules.

Based on the above discussion, in linear PBAT, water molecules gradually eroded the material, forming microchannels or micropores, a process facilitated by the hydrophilic monomers generated during degradation. In contrast, in branched PBAT, the branched units hindered the penetration of water molecules. As a result, compared to linear PBAT, the probability of water molecules dissolving in the PBAT phase region was reduced, thereby inhibiting degradation.

### Rheological Property

The influence of the branched structure on the polymer melt was further studied. Given that the molecular weights were similar for all samples (Table 2), it was reasonable to attribute differences in rheological properties to variations in chain structure. Small-amplitude oscillatory shear (SAOS) tests were conducted at 160 °C to analyze the relaxation behavior of the polymer chains in the PBAT copolymers. The Maxwell model^54^ was applied to fit the small-amplitude oscillatory shear data using eqs 18 and 19, where *g*_*i*_ represents the modulus contribution of each model element and τ_i_ is the relaxation time associated with it.
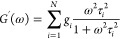
18
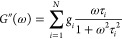
19
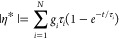
20

The relaxation spectrum *g*_*i*_ and τ_*i*_ could be obtained through fitting (as shown in Table S4) and used to determine the zero-shear viscosity η_0_ by extrapolating ω to zero, as indicated in eq 20. Additionally, the Carreau–Yasuda model was also used to determine η_0_ and the shear-thinning index due to the approximate Newtonian plateau observed in the low-frequency region during the SAOS test.
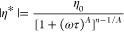
21

**Figure 11:**
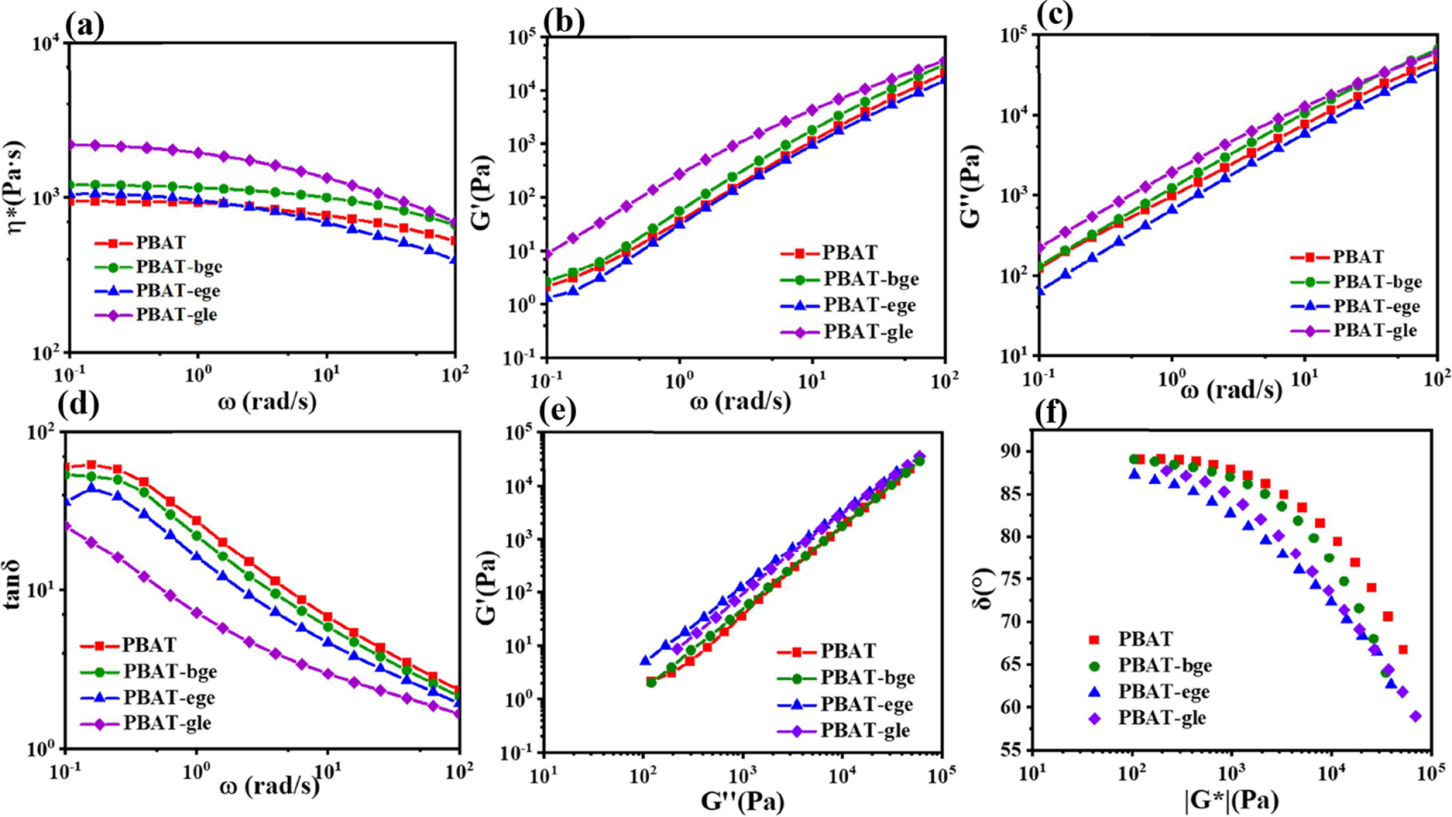
Complex viscosity η* (a), loss factor tanδ (b), storage modulus *G*′(c), loss modulus *G*″ (d), Han cure (e), and Van Gurp–Palmen (f) of linear and branched PBAT samples at 160 °C.

Given their similar molecular weights, branched PBAT-bge and PBAT-gle exhibited higher complex viscosities than linear PBAT at low frequencies (Table 4). The approximate Newtonian plateau observed in Figure 11a suggests that chain entanglements had sufficient time to reform. The increased viscosity in branched PBATs could be attributed to more significant entanglements between the branches and main chains. As frequency increased, all PBAT copolymers exhibited shear thinning, with branched variants showing a more pronounced effect. This was reflected in their lower shear-thinning index, decreasing from 1.41 to 1.25 in the terminal region, which arose from the branched architecture.

**Table 4:** Rheological Property of Linear and Branched PBAT Samples

sample	η_0_ (Pa · s)^a^	η_0_ (Pa · s)^b^	λ (s)	*n*	*A*	slope
PBAT	850.3	958.2	0.18	1.41	0.88	1.28
PBAT-bge	1200.1	1227.7	0.23	1.37	0.72	1.31
PBAT-ege	957.1	1082.6	0.44	1.25	0.89	1.16
PBAT-gle	1942.5	2290.9	0.23	1.28	0.79	1.49

a The zero-shear viscosity from the Maxwell model.

b The zero-shear viscosity from the Carreau–Yasuda model.

In block copolymer melts with disordered phase domains, the storage modulus *G′* typically exhibits a slope ranging from 1.0 to 1.5.^55^ For PBAT, the presence of long sequences of BT blocks, BA blocks, and random BA-BT segments results in behavior similar to that of multiblock copolymers. As illustrated in Figure 11b,e, the low-frequency slope of PBAT shows a gradual deviation from 2, lacking the characteristics of ordered phases. This observation suggests the potential existence of unordered domain morphology within the PBAT melts.

The multistep relaxation behavior resulting from the incorporation of branched chains was further validated by the tanδ-frequency (tanδ−ω) curves. The loss factor tanδ was defined as the ratio of *G″* to *G*′, representing the transition from viscous to elastic responses in the polymer melt. Throughout the entire frequency range, the loss modulus consistently exceeded the storage modulus (tanδ > 1), indicating that viscous dissipation dominated the melt behavior. The introduction of branched chains increased the elastic response of the melt, which was reflected in a lower tanδ at the same frequency (tanδ_branched_ < tan δ_linear_).

The Han plot was used to visualize the relationship between *G″* and *G′*, offering deeper insights into the viscoelastic properties. According to the Masuda and Han model, the shape of the Han curve was independent of temperature and molecular weight.^56^ The introduction of branching units enhanced chain entanglements, which, in turn, restricted chain mobility, resulting in slower relaxation dynamics and a reduced slope. Even a low content of branching significantly enhanced the melt elasticity and contributed to a more “solid-like” behavior.

The Van Gurp–Palmen (vGP) curves for branched and linear PBAT (Figure 11f) overlapped well at low |G*|, indicating similar long-chain branching and relaxation behaviors. However, the Van Gurp–Palmen curves did not exhibit a minimum in the vGP plot, suggesting a distribution of the relaxation modes. At higher |G*| values, the introduction of branched units caused the curve to shift slightly to the left, with an inflection point at a higher phase angle. This shift likely resulted from the increased complexity of relaxation processes due to slower dynamics (Table S4) and entanglements between branches and the main chain, a trend also observed in branched polyethylene. The verification of the film-blowing processability section is discussed in the Supporting Information.

## Conclusion

Branched and linear PBAT copolymers with similar molecular weights were successfully synthesized by using a two-step method. Comprehensive studies were conducted on the hierarchical structure of branch unit chains and their effects on biodegradation properties, as well as thermal and rheological properties. Based on the proportion of linear and branch units in ^1^H-NMR, the “Memoryless Random Model” was proposed to calculate the continuous sequence distribution of BT and BA. The conformational parameters *g* and *B*_3*w*_ were determined by gel permeation chromatography coupled with triple detectors, thereby providing a clear definition of the branching characteristics. The introduction of branch units led to a gradual decrease in the crystallization temperature and a reduction in crystallinity from 20.83% (linear PBAT) to 14.36% (branched PBAT). FTIR combined with XRD analysis revealed two crystalline forms in PBAT, with the β-form predominating in linear PBAT. Enzymatic degradation studies showed a reduced degradation rate for branched PBAT compared to linear PBAT, with distinct differences in degradation fragments. This reduction in degradation rate was attributed to the decreased probability of water molecules dissolving in the branched PBAT phase region and the enhanced hydrophobicity. The copolymers exhibited higher complex viscosity and tanδ at the same frequency, indicating an enhanced melt elastic response. The deviation of the end slope in the Han curve also confirmed the existence of BA-BT random block sequences and complex relaxation behavior. In this work, the branched PBAT polyester with a new structure has improved the melt strength, which is important to improving the processing film blowing stability. It can be expected to expand the application range of PBAT, especially for agricultural mulching films.

## Supporting Information Available

Molecular structure and physical property of branch units; Original FTIR, ^1^H-NMR spectra; GPC curves; Calculation for concertration of cutinase; Calculation for transfer matrix “Memoryless Random Model” Degradation products identification by LC/MS; Water contact angle test; Surface morphology and SEM image of PBAT film during enzymatic hydrolysis; Chemical composition and the model of PBAT-0 and PBAT-graft; Sections about mechanical property; and verification of film-blowing processability (PDF)
