# Supplementary material for: Synthesis of Poly(butylene adipate-co-terephthalate) with Branched Monomer for Biodegradable Copolyesters with Enhanced Processability and Rheological Properties
Source: ACS Omega. 2025 Apr 4;10(14):14258–70. doi: 10.1021/acsomega.5c00277 (PMC12004134; doi:10.1021/acsomega.5c00277)
Supplement: Supplementary file 1 — ao5c00277_si_001.pdf [file ao5c00277_si_001.pdf]

Supporting information

Synthesis of Poly (butylene adipate-co-terephthalate) with Branched Monomer for Biodegradable Copolyesters with Enhanced Processability and Rheological Properties

Xinpeng Zhang[1], Hongli Bian[1], Xiangze Meng[1], Jing Yuan[1], Jianping Ding[2], Wanli Li[2], Jun Xu[1*], Baohua Guo[1*]

[1]Key Laboratory of Advanced Materials (MOE), Department of Chemical Engineering, Tsinghua University, Beijing 100084, China

[2] Xinjiang Blue Ridge Tunhe Sci. & Tech. Co., Ltd. Changji 831199, Xinjiang, China

*Correspondence: jun-xu@mail.tsinghua.edu.cn

*Correspondence: bhguo@mail.tsinghua.edu.cn

Number of Pages: 19

Number of Figures: 12

Number of Tables: 4

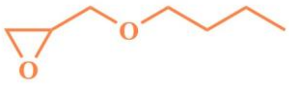

Butyl glycidyl ether

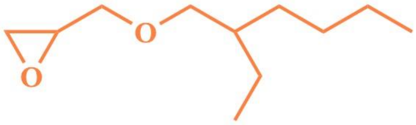

Ethylhexyl glycidyl ether

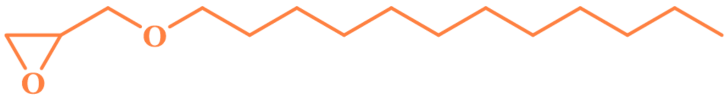

Glycidyl lauryl ether

Figure S1. Molecular structure of branching monomer.

Table S1. Physical Properies Information of Branching Monomer

Branched monomer	Molecular formula	Molecular weight	Density	Boiling point(at 760 mmHg)
Butyl glycidyl ether	$C_7H_{14}O_2$	130.18 g/mol	0.91 g/cm³	177.0 ℃
Ethylhexyl glycidyl ether	$C_{11}H_{22}O_2$	186.29 g/mol	0.89 g/cm³	259.4 ℃
Glycidyl lauryl ether	$C_{15}H_{30}O_2$	242.4 g/mol	0.90 g/cm³	303.1℃

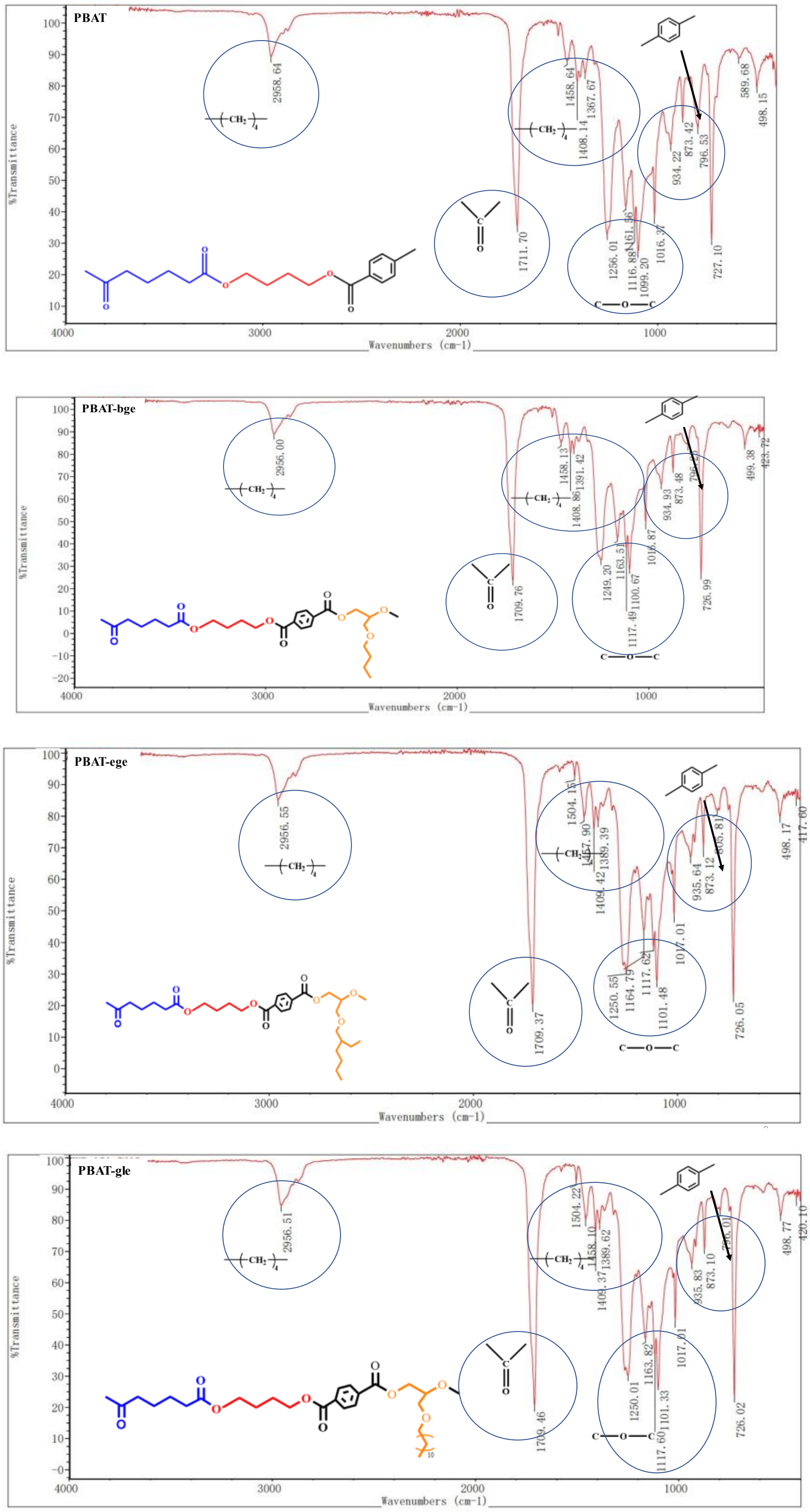

Figure S2. Original FTIR spectra of the linear and branched PBAT copolymers.

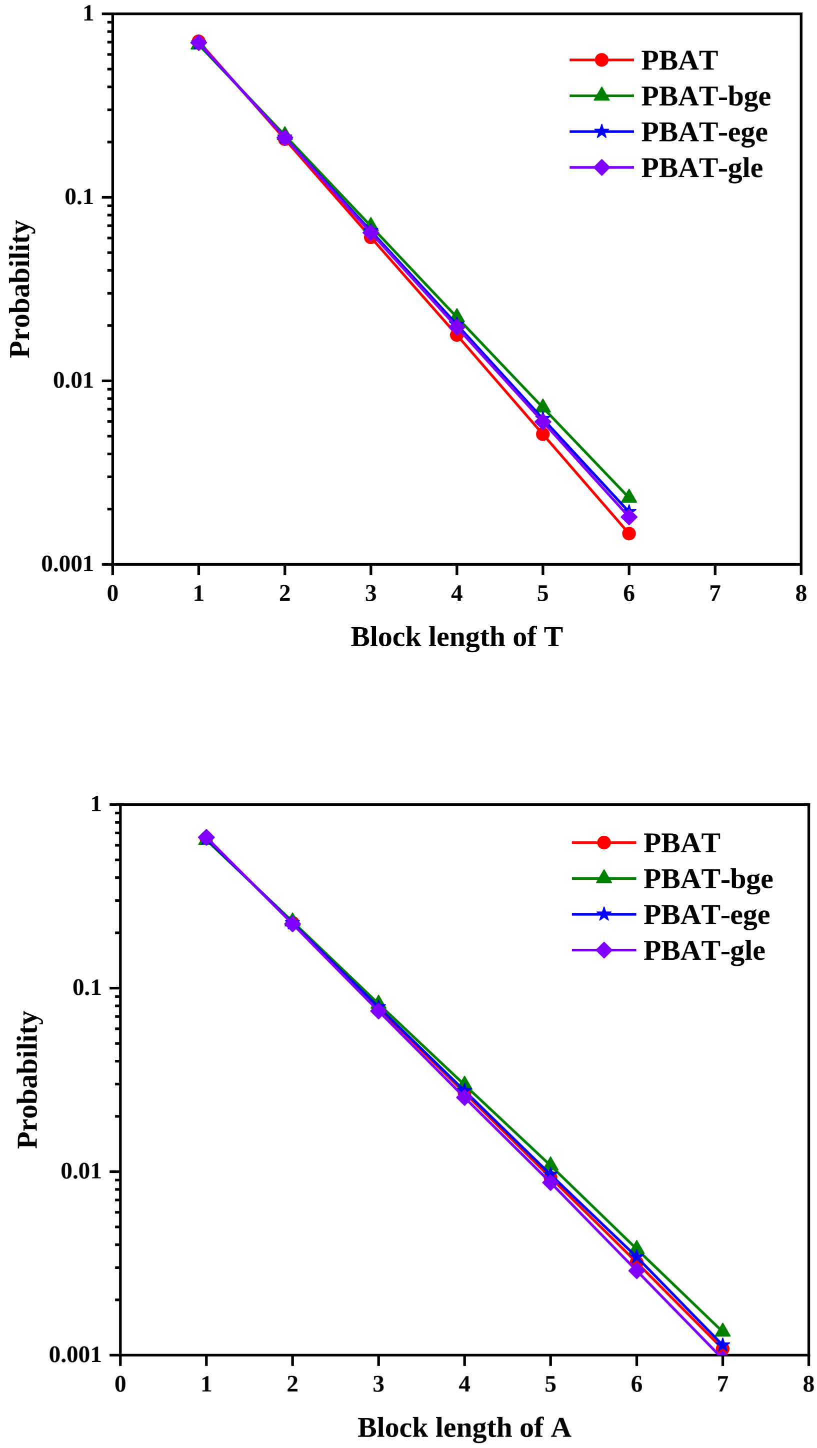

Figure S3. The probability of the consecutive blocks in linear and branched PBAT copolymers.

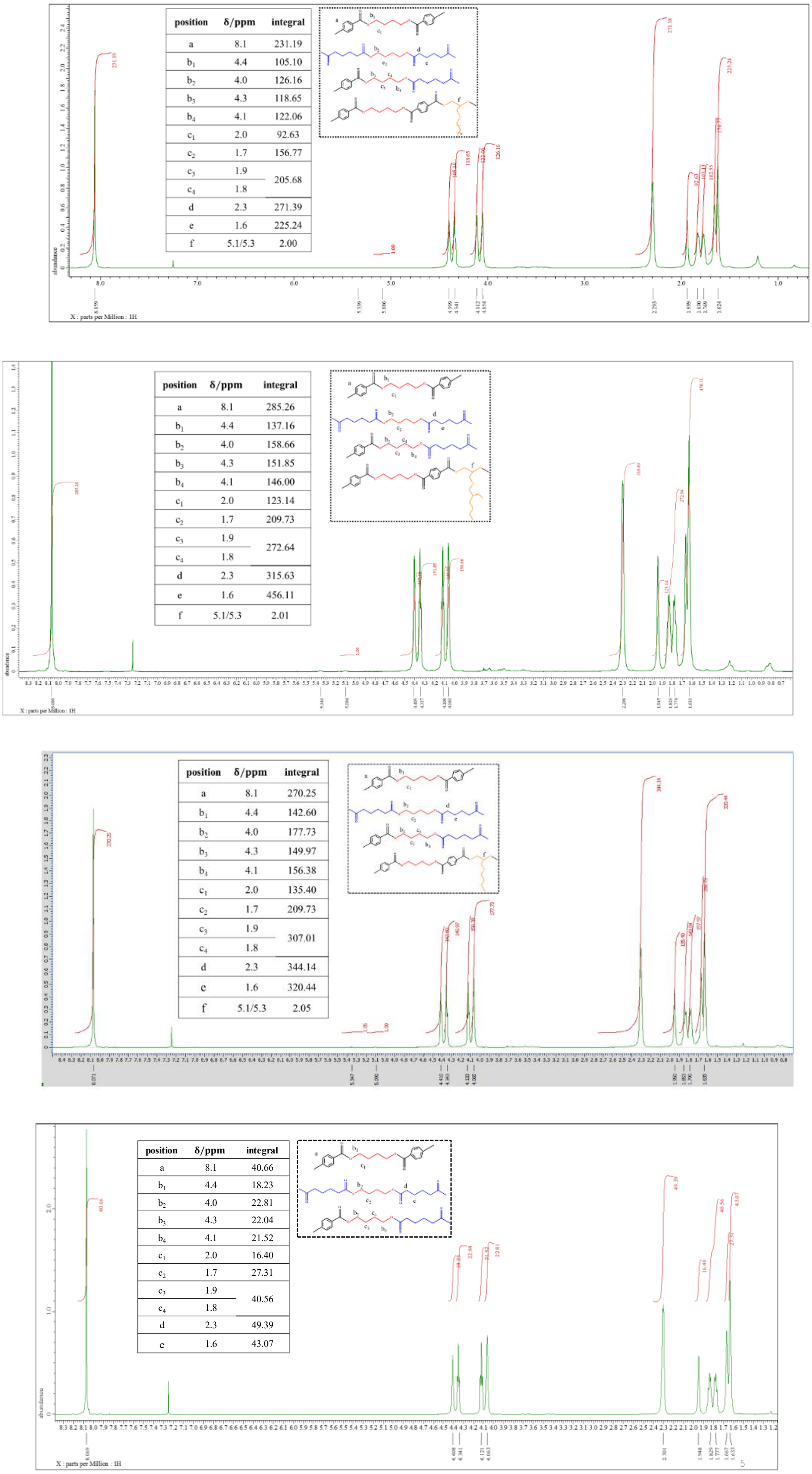

Figure S4. Original ^{1}H-NMR spectra of the linear and branched PBAT copolymers.

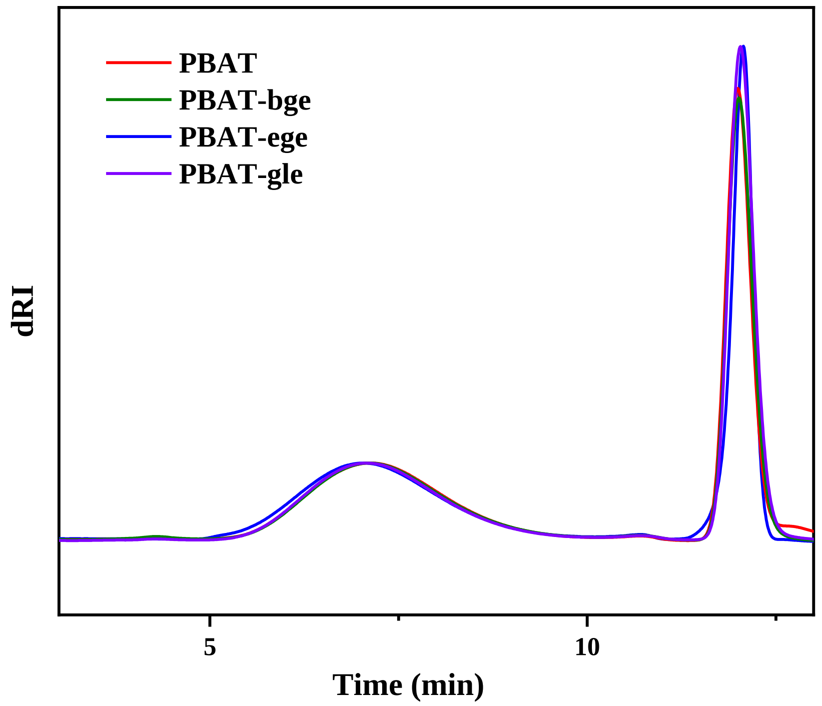

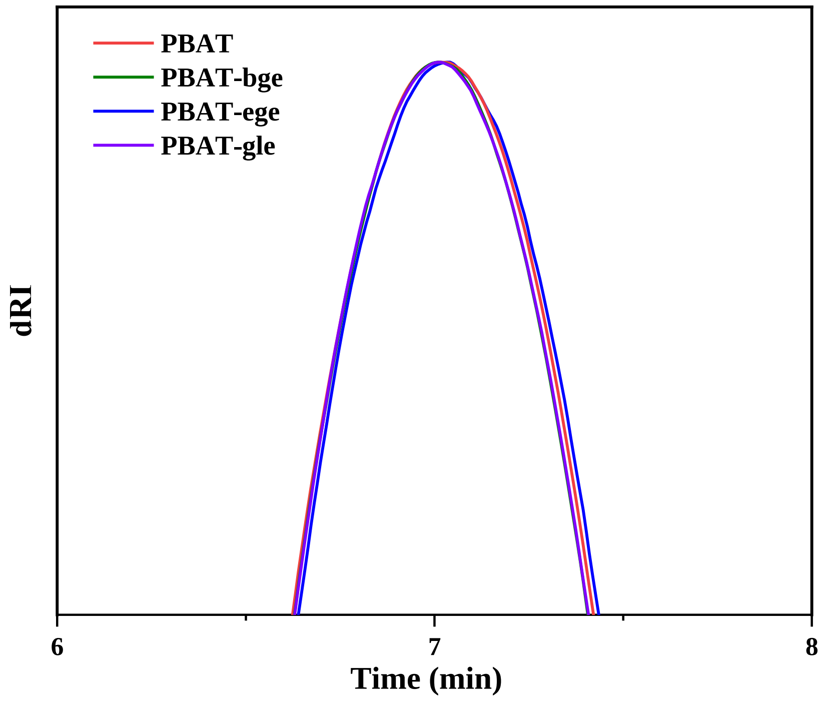

Figure S5. GPC curves of the linear and branched PBAT copolymers.

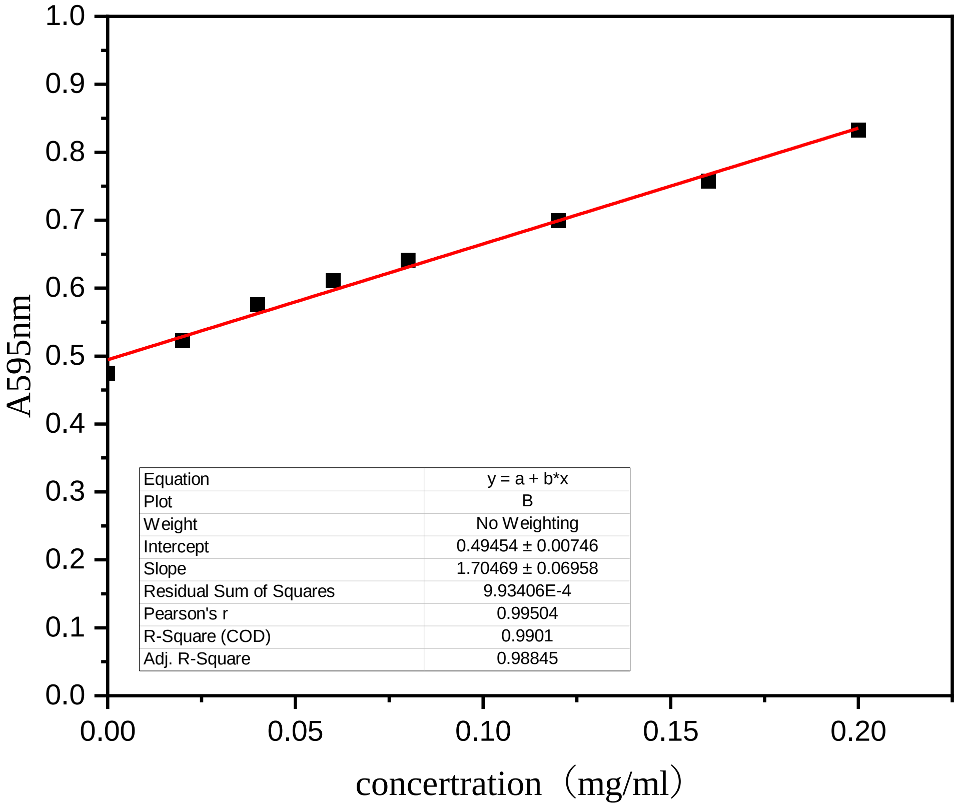

Figure S6. microplate reader method calculation for the concentration of Novozym51032.

$$\tilde{y} = 1.705x + 0.4945 \quad (R^2 = 0.9901)$$

Diluted 10 times:

$$y = \frac{0.5855 + 0.5984 + 0.6122}{3} = 0.5987 \Longrightarrow x{=}0.06$$

Diluted 5 times:

$$y = \frac{0.6949 + 0.6982 + 0.7057}{3} = 0.6996 \Longrightarrow x{=}0.12$$

The concentration of Novozym51032 is 0.6mg/ml

Number	Charge mass ratio	Retention time	Formula	Proposed structure	note
1	90.06	8.72	$C_4H_{10}O_2$	B	
2	122.03	9.75	$C_7H_6O_2$	Bz	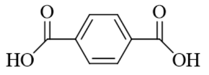
3	138.03	9.75	$C_7H_6O_3$	HBA	
4	146.05	7.55	$C_6H_{10}O_4$	A	Terephthalic acid ($C_8H_6O_4$-166)
5	165.01	9.75	$C_8H_6O_4$	T	
6	218.11	13.38	$C_{10}H_{18}O_5$	AB	
7	238.10	13.32	$C_{12}H_{14}O_5$	TB	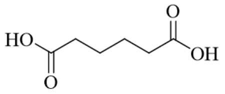
8	290.17	18.78	$C_{14}H_{26}O_6$	BAB	Adipate acid ($C_6H_{10}O_4$-146)
9	310.28	16.63	$C_{16}H_{22}O_6$	BTB	
10	366.22	18.49	$C_{18}H_{22}O_8$	ABT	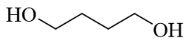
11	418.22	18.07	$C_{20}H_{34}O_9$	BABA	
12	438.19	21.73	$C_{22}H_{30}O_9$	BABT	Butanediol ($C_4H_{10}O_2$-90)
13	458.15	23.21	$C_{24}H_{26}O_9$	BTBT	
14	490.27	20.10	$C_{24}H_{42}O_{10}$	BABAB	
15	510.96	21.10	$C_{26}H_{38}O_{10}$	BABTB	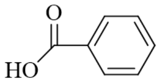
16	530.22	24.35	$C_{28}H_{34}O_{10}$	BTBTB	
17	586.45	26.14	$C_{30}H_{34}O_{12}$	ABTBT	Benzonic acid ($C_7H_6O_2$-122)
18	606.17	25.34	$C_{32}H_{30}O_{12}$	TBTBT	
19	638.29	24.59	$C_{32}H_{46}O_{13}$	BABABT	
20	678.23	25.89	$C_{36}H_{38}O_{13}$	BTBTBT	

Figure S7. Degradation products identified by LC/MS during PBAT biodegradation.

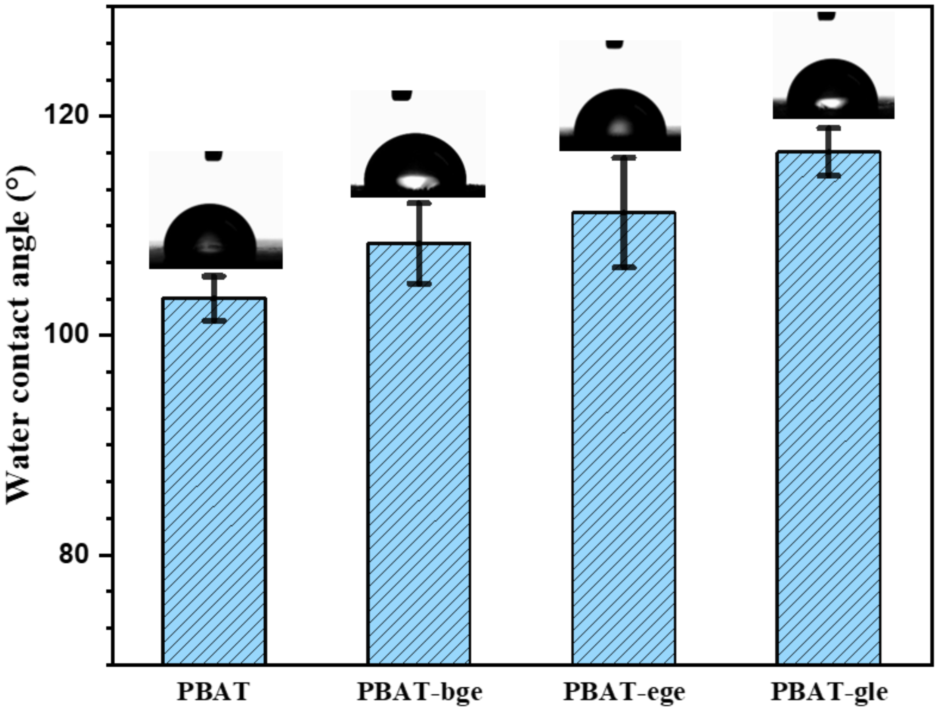

Figure S8. Water contact angle test of linear and branched PBAT.

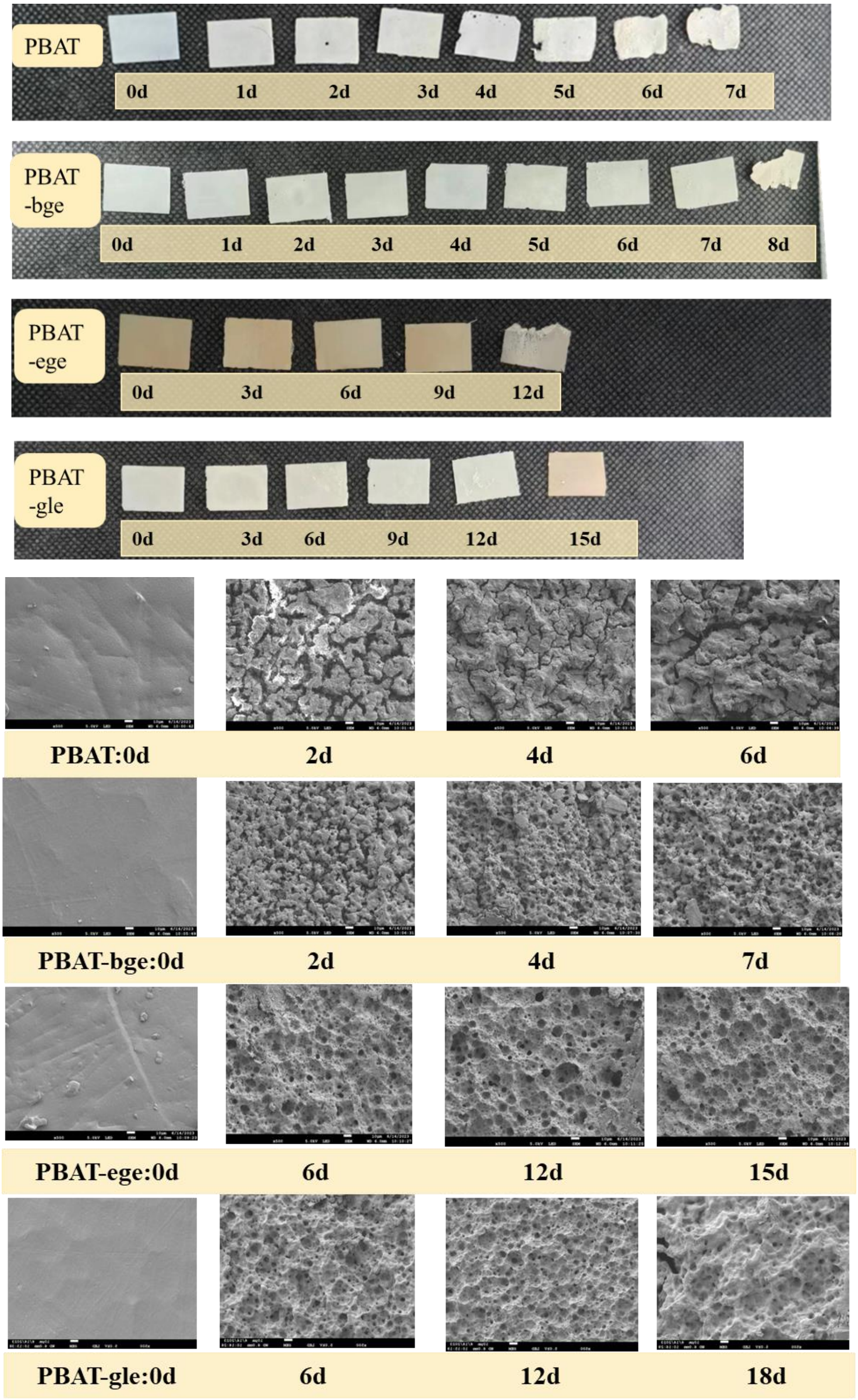

Figure S9. Surface morphology and SEM image of PBAT film during enzymatic hydrolysis.

DFT Calculations of Interaction Enthalpy (ΔH$_{mix}$) Between Water Molecules and PBAT Units

Density Functional Theory (DFT) calculations were performed to investigate the ΔH_{mix} between water molecules and specific PBAT units, PBAT segments, and branching units. ΔH_{mix} is defined as:

$$\Delta H_{mix} = H_{complex} - H_{H_2O} - H_X$$

where X represents PBAT units, PBAT segments, or branching units, PBAT units refer to the monomer used to synthesize non-branched PBAT, including 1,4-BDO, AA and PTA, which is also the product of complete hydrolysis of non-branched PBAT. PBAT segments refer to the segments on the PBAT molecular chain, whether branched or not, including the corresponding segments of 1,4-BDO, AA and PTA after polycondensation. Branching units refers to branching units, including bge, ege and gle. $H_{complex}$ corresponds to the enthalpy of the complex formed between X and water molecules. A smaller value of ΔH_{mix} indicates greater hydrophilicity of X, suggesting that water molecules are more likely to penetrate the structure.

The DFT calculations utilized the M06-2X functional, [1, 2]which is widely applied in studies of organic systems.[3-5] Geometric optimizations and vibrational analyses were conducted using the 6-31G** basis set, while single-point energy calculations employed the jul-cc-PVTZ basis set due to the need for higher precision in describing intermolecular interactions. A zero-point energy (ZPE) correction factor of 0.97 was applied.[6, 7]All geometrically optimized models were verified to exhibit no imaginary frequencies.

MD Simulations of Water Diffusion Behavior in PBAT

Molecular Dynamics (MD) simulations were performed to investigate the water diffusion behavior in random copolymer PBAT models, including a linear PBAT model (PBAT-0) and a branched PBAT model (PBAT-graft). The chemical compositions of these models are summarized in Figure S11. The COMPASS II force field, which is well-suited for polymer systems, was employed for the simulations.[7-9]

The models were first subjected to initial geometric optimization, followed by 0.5 ns of annealing relaxation under the NPT ensemble (298.15–2000 K, five cycles) and 0.5 ns of isothermal relaxation at 298.15 K. The density and energy of the final relaxed models fluctuated around stable values, indicating that the models had reached equilibrium. The final optimized structures of PBAT-0 and PBAT-graft are shown in Figure S10.

Subsequently, 0.5 ns of isothermal relaxation at 298.15 K was conducted to obtain the Mean Squared Displacement (MSD)-Time curves for water molecules. Diffusion coefficients were calculated using Einstein's equation based on the linear regions of the MSD-Time curves. The diffusion coefficients of water in PBAT-0 and PBAT-graft are denoted as $D_{H_2O\,-PBAT-0}$ and $D_{H_2O\,-PBAT-graft}$, respectively.

Name	B	A	T	DGE	H_2O
PBAT-0	100%	55%	45%	0%	100%
PBAT-graft	90%	45%	45%	10%	100%

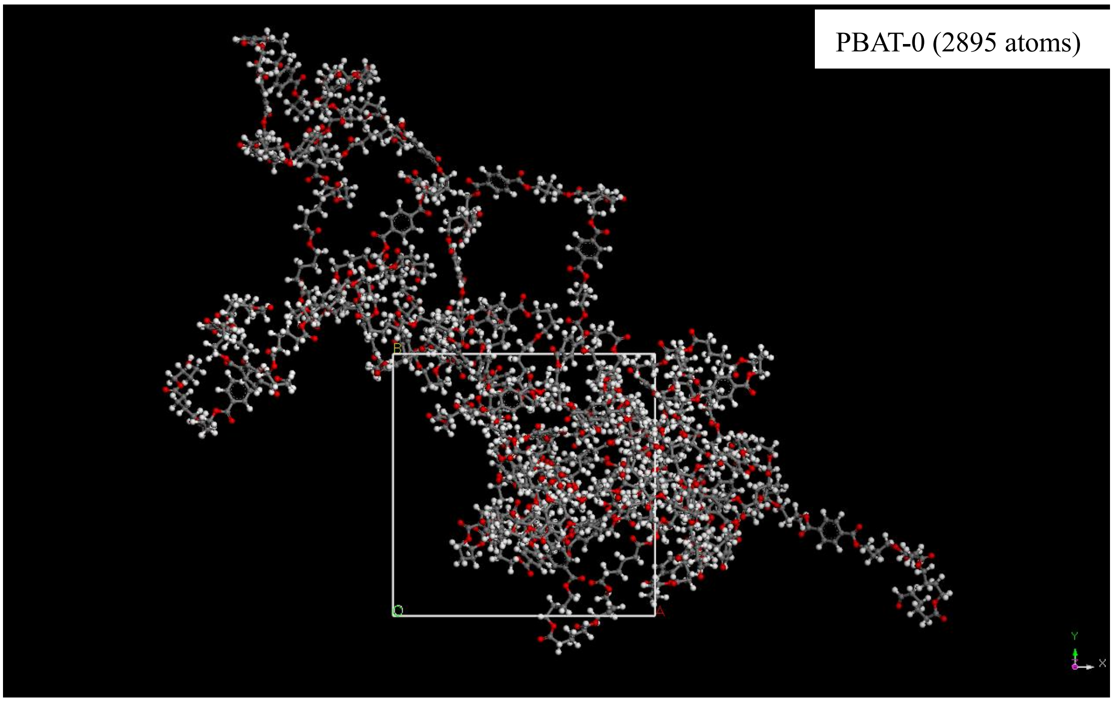

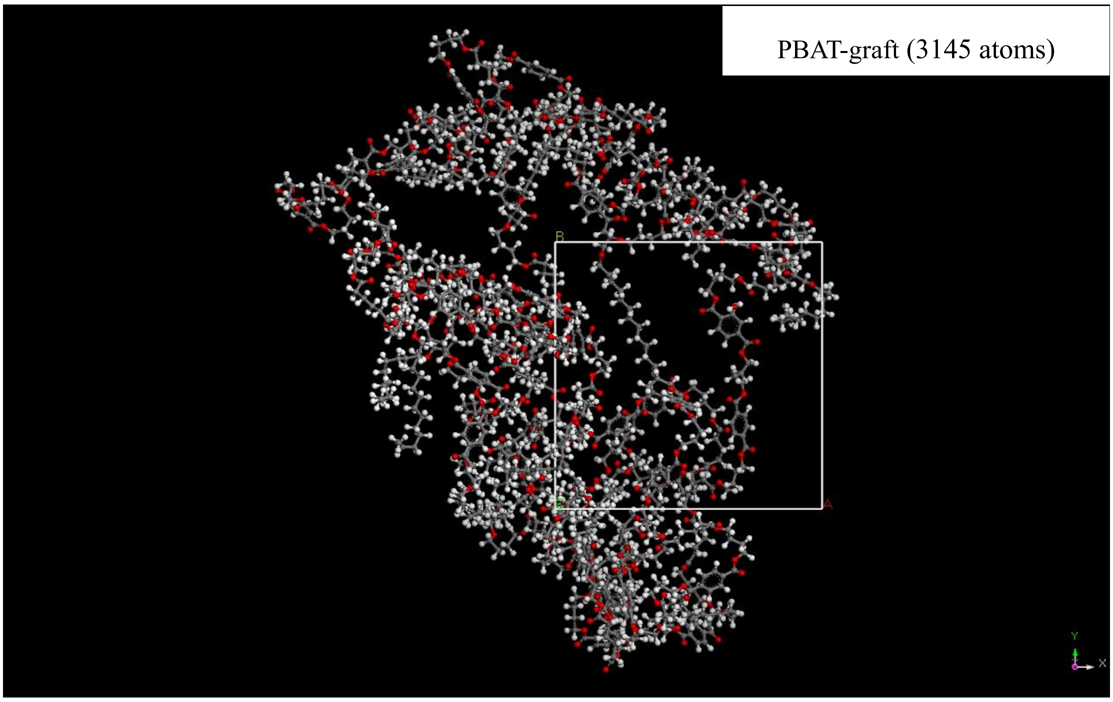

Figure S10. The chemical composition and the model of PBAT-0 and PBAT-graft.

Calculation of transfer matrix "Memoryless Random Model"

To deeply understand such differences and their impact on the microstructures of these branched-PBAT, a detailed ^{1}H-NMR analysis of the main chain was conducted. The probability that the branched units is next to the linear units (P_{A-br} and P_{T-br}), the probability that linear units is next to the branched units containing (P_{br-A} and P_{br-T}) and the probability that linear units is next to the linear units (P_{T-T}, P_{T-A}, $P_{A-A,}$ $P_{A-T,}$) are calculated by below eqs1-8. There should be noted that the probability that branched units is next to the linear units $P_{br-br} = 0$. That is determined by the reaction mechanism of the added branched monomer. In the reaction system, there will be no two continuous epoxy functions participating in the reaction. Therefore, there will be no two continuous branched units.

$$P_{T-T} = \frac{I_{TBT}}{I_{TBT} + 2I_{TBA} + 4I_{TGT} + 4I_{TGA} + 4I_{TGB}} \quad (1)$$

$$P_{T-A} = \frac{2I_{TBA}}{I_{TBT} + 2I_{TBA} + 4I_{TGT} + 4I_{TGA} + 4I_{TGB}} \quad (2)$$

$$P_{T-br} = \frac{4I_{TGT} + 4I_{TGA} + 4I_{TGB}}{I_{TBT} + 2I_{TBA} + 4I_{TGT} + 4I_{TGA} + 4I_{TGB}} \quad (3)$$

$$P_{A-A} = \frac{I_{ABA}}{I_{ABA} + 2I_{ABT} + 4I_{AGT} + 4I_{AGA} + 4I_{AGB}} \quad (4)$$

$$P_{A-T} = \frac{2I_{ABT}}{I_{ABA} + 2I_{ABT} + 4I_{AGT} + 4I_{AGA} + 4I_{AGB}} \quad (5)$$

$$P_{A-br} = \frac{4I_{AGT} + 4I_{AGA} + 4I_{AGB}}{I_{ABA} + 2I_{ABT} + 4I_{AGT} + 4I_{AGA} + 4I_{AGB}} \quad (6)$$

$$P_{br-A} = \frac{I_{TGA} + I_{AGA} + I_{TGB} + I_{AGB}}{I_{TGT} + I_{TGA} + 2I_{TGB} + I_{AGT} + I_{AGA} + 2I_{AGB}} \quad (7)$$

$$P_{br-T} = \frac{I_{AGT} + I_{AGA} + I_{TGB} + I_{AGB}}{I_{TGT} + I_{TGA} + 2I_{TGB} + I_{AGT} + I_{AGA} + 2I_{AGB}} \quad (8)$$

Verification of Film-Blowing Processibility

To verify the improvement in the melt elastic response of branched PBAT, we conducted scale-up synthesis in a reaction kettle and produced batches of both linear and branched PBAT for small-scale film-blowing tests.

The entrance pressure drop occurs due to the strong tensile effect caused by the convergent flow of the melt as it enters the die from the capillary barrel. This process increases the flow rate and stretches the chain segments, stores energy, and reduces pressure. A larger entrance pressure drop indicates stronger elastic deformation of the chain during the tensile motion of the chain segments, which promotes tensile hardening. The tensile hardening is beneficial for achieving the uniform film thickness.

Notably, the entrance pressure drop differs significantly between the two chain structures of PBAT. As shown in Figure S11a, linear PBAT, which exhibits a lower entrance pressure drop, has poor film-blowing uniformity. In contrast, branched PBAT, with a higher entrance pressure drop, can be easily blown into a uniform film. This observation is consistent with the rheological property analysis discussed above.

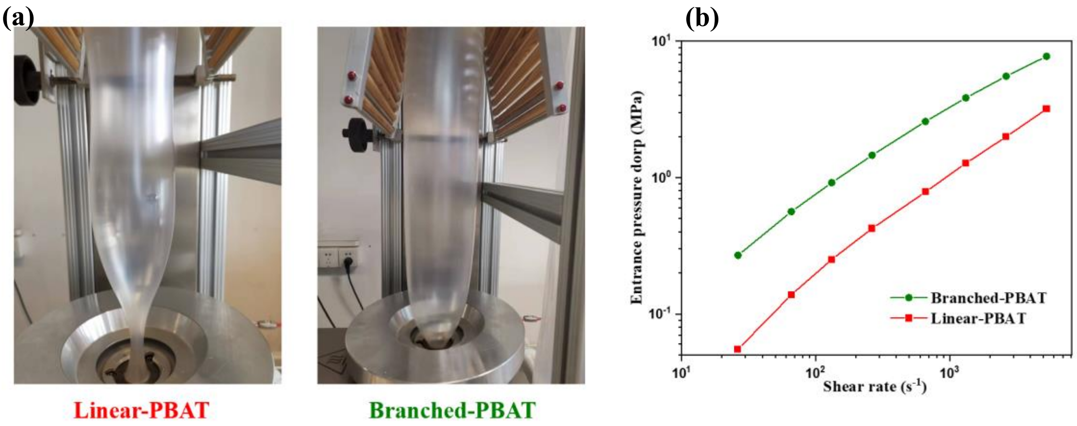

Figure S11. Film-blowing experiments of linear and branched PBAT(a) and entrance pressure drop test (b).

Mechanical Property

The mechanical properties of polymers are critical parameters for their practical applications. In this study, the effect of different branch chain lengths on the mechanical properties of linear PBAT was investigated by designing the molecular chain structure and introducing branch units into the main chain. Figure S12 presents the stress−strain curves of PBAT copolymers. The elastic modulus, stress and elongation at break are summarized in Table S2.

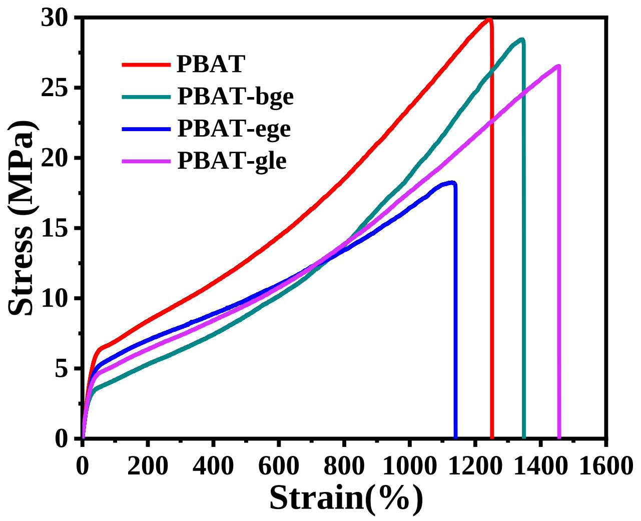

Figure S12. Stress−strain curves of linear and branched PBAT samples.

The PBAT copolyesters exhibit excellent elasticity and flexibility. The crystallized BT hard segments contribute to the strength of the copolymers, while the soft BA segments impart flexibility. The stress-strain curves of PBAT copolymers display typical plastic behavior with significant elongation at break. Additionally, the copolymers exhibit pronounced strain hardening, attributed to strain-induced crystallization and chain alignment.

Table S2. Mechanical Property of Linear and Branched PBAT Samples

sample	E(MPa)	ε_b(%)	σ_b(MPa)
PBAT	20.6±2.1	1246±65	29.8±1.6
PBAT-bge	28.9±2.6	1343±68	27.3±1.2
PBAT-ege	22.3±2.2	1091±80	16.8±1.8
PBAT-gle	24.3±1.7	1213±72	25.8±1.4

Since PBAT copolymer samples had similar molecular weights, the degrees of crystallinity and the chemical compositions were the primary factors influencing their mechanical properties. Increasing crystallinity enhanced the modulus but reduced the strain at break. In this study, linear PBAT exhibited the highest tensile strength (29.8 MPa), though its break elongation (1246%) was lower than that of PBAT-bge. This variation is attributed to differences in crystallinity among the copolymers. The introduction of branch units disrupted the rigidity and regularity of the PBT segments, leading to a decrease in crystallinity.

Table S3. Normalization of the Area Proportions and Wavenumber

sample	Peak1	Area(%)	Peak2	Area(%)	Peak3	Area(%)
PBAT	1700	21	1710	32	1728	47
PBAT-bge	1693	11	1708	31	1725	58
PBAT-ege	1694	12	1708	29	1725	59
PBAT-gle	1694	11	1708	24	1724	65

Table S4. Relaxation Spectra of PBAT defined by RepTate software

PBAT		PBAT-bge		PBAT-ege		PBAT-gle	
g	τ	g	τ	g	τ	g	τ
4.62E+06	5.13E-05	2.57E+00	1.97E+01	3.93E-06	1.10E+01	3.85E+06	7.80E-05
3.36E+00	2.60E-04	1.06E-06	6.33E+00	1.50E-05	3.34E+00	1.11E-03	3.67E-04
8.44E-07	1.31E-03	2.87E-06	2.03E+00	1.12E+02	1.02E+00	1.09E-03	1.73E-03
5.02E+04	6.65E-03	6.52E+01	6.52E-01	2.63E+02	3.10E-01	5.46E+04	8.13E-03
5.04E+03	3.37E-02	3.71E+02	2.09E-01	2.39E+03	9.42E-02	1.15E+04	3.83E-02
5.84E+02	1.70E-01	2.31E+03	6.71E-02	3.17E+03	2.87E-02	2.87E+03	1.80E-01
1.44E-06	8.62E-01	1.03E+04	2.15E-02	2.77E+04	8.73E-03	2.84E+02	8.48E-01
1.07E-10	4.36E+00	5.08E+04	6.91E-03	2.09E-04	2.66E-03	4.03E-05	3.99E+00
2.56E-07	2.21E+01	1.93E-01	2.22E-03	1.47E-02	8.08E-04	1.93E-05	1.88E+01
3.15E-08	1.12E+02	5.38E+05	7.11E-04	8.24E+05	2.46E-04	1.29E-05	8.84E+01

References

1. Zhao, Y.; Truhlar, D. G., The M06 suite of density functionals for main group thermochemistry, thermochemical kinetics, noncovalent interactions, excited states, and transition elements: two new functionals and systematic testing of four M06 functionals and 12 other functionals. Theoretical Chemistry Accounts 2008, 119 (5), 525-525.
2. Zhao, Y.; Truhlar, D. G., Density Functionals with Broad Applicability in Chemistry. Accounts of Chemical Research 2008, 41 (2), 157-167.
3. Paytakov, G.; Dinadayalane, T.; Leszczynski, J., Toward Selection of Efficient Density Functionals for van der Waals Molecular Complexes: Comparative Study of C–H···π and N–H···π Interactions. The Journal of Physical Chemistry A 2015, 119 (7), 1190-1200.
4. Olsen, S. T.; Elm, J.; Storm, F. E.; Gejl, A. N.; Hansen, A. S.; Hansen, M. H.; Nikolajsen, J. R.; Nielsen, M. B.; Kjaergaard, H. G.; Mikkelsen, K. V., Computational Methodology Study of the Optical and Thermochemical Properties of a Molecular Photoswitch. The Journal of Physical Chemistry A 2015, 119 (5), 896-904.
5. Breugst, M.; Eschenmoser, A.; Houk, K. N., Theoretical Exploration of the Mechanism of Riboflavin Formation from 6,7-Dimethyl-8-ribityllumazine: Nucleophilic Catalysis, Hydride Transfer, Hydrogen Atom Transfer, or Nucleophilic Addition? Journal of the American Chemical Society 2013, 135 (17), 6658-6668.
6. Papajak, E.; Truhlar, D. G., Efficient Diffuse Basis Sets for Density Functional Theory. Journal of Chemical Theory and Computation 2010, 6 (3), 597-601.
7. Alecu, I. M.; Zheng, J.; Zhao, Y.; Truhlar, D. G., Computational Thermochemistry: Scale Factor Databases and Scale Factors for Vibrational Frequencies Obtained from Electronic Model Chemistries. Journal of Chemical Theory and Computation 2010, 6 (9), 2872-2887.
8. Padrela, L.; Zeglinski, J.; Ryan, K. M., Insight into the Role of Additives in Controlling Polymorphic Outcome: A CO2-Antisolvent Crystallization Process of Carbamazepine. Crystal Growth & Design 2017, 17 (9), 4544-4553.
9. Guo, S.; Wu, Y.; Luo, S.-X. L.; Swager, T. M., Versatile Nanoporous Organic Polymer Catalyst for the Size-Selective Suzuki–Miyaura Coupling Reaction. ACS Applied Nano Materials 2022, 5 (12), 18603-18611.
